# Resolving the heterogeneity of dopamine subsystems dysfunction in schizophrenia: a PET meta-analysis

**DOI:** 10.1038/s41537-025-00684-0

**Published:** 2025-11-21

**Authors:** Zhen Zhao, Xin Li, Yingying Xie, Liyuan Lin, Luli Wei, Zhongyu Chang, Yun Luo, Haoyang Dong, Xue Zhang, Qiqi Dong, Chunshui Yu, Meng Liang, Hao Ding, Wen Qin

**Affiliations:** 1 Department of Radiology, Tianjin Key Lab of Functional Imaging, Tianjin Institute of Radiology and State Key Laboratory of Experimental Hematology, Tianjin Medical University General Hospital, Tianjin, China; 2 School of Medical Imaging, Tianjin Medical University, Tianjin, China

**Keywords:** Schizophrenia, Molecular neuroscience, Biomarkers

## Abstract

The “dopamine hypothesis” of schizophrenia suggests the imbalance of the brain dopamine system plays a crucial role in the development of this disorder. Although this hypothesis has been partly supported by early studies, the brain region-specific abnormalities of different dopamine subsystems, and the influential factors on the heterogeneity of dopamine dysfunction of schizophrenia are still unknown. To address these issues, we carried out random-effect meta-analyses by collecting 49 in vivo PET studies (692 patients and 730 controls). Patients exhibited significant regional and population heterogeneity in D_2/3_ receptor availability, primarily manifesting as a moderate increased in the striatum of only drug-off patients (Cohen’s d = 0.56, 95%CI [0.16; 0.97]), whereas only drug-on patients showed a large decrease in D_2/3_ receptor availability in the thalamus, limbic lobe, substantia nigra, and temporal lobe (Cohen’s d < −1.40). Drug-off patients also had a small increase in dopamine synthesis in the striatum (Cohen’s d = 0.38, 95%CI[0.06; 0.69]), with no replicable dysfunctions in drug-on patients or other brain regions. D_1_ receptor availability and DAT showed high heterogeneity but no consistent significances. Finally, Meta-regression indicated that elevated striatal D_2/3_ levels correlated with a higher proportion of males, older patients, longer duration and worse symptoms predicted more severe D_1_ deficits in the striatum and prefrontal cortex. This study suggests that different dopamine subsystems in schizophrenia are selectively involved across brain regions; moreover, medication status, sex, age, illness duration and symptom diversity have significant impacts on the dysfunctions of dopamine subsystems in schizophrenia.

## Introduction

The “dopamine hypothesis” is one of the leading pathoetiologic theories of schizophrenia and has undergone significant evolution since its inception^1–3^. The classical hypothesis was originally proposed in the mid-20th century following the discovery of antipsychotic drugs dopamine D_2_ receptor antagonists primarily binding the striatum, thus linking D_2_ receptor increased in the striatum to positive symptoms like hallucinations and delusions^4^. However, in recent decades, advancements in understanding dopamine functional subsystems shifted attention to extra-striatal regions such as the mesocortical and mesolimbic pathways, which proposed that dopamine deficit in these pathways, particularly involving the D_1_ receptor, could better explain the negative symptoms such as social withdrawal and cognitive impairment of schizophrenia^5^. Moreover, recent multidimensional evidence proposed that schizophrenia arises from a complex interplay between dopamine systems and other neurotransmitters that are complexly influenced by genetic, environmental, and neurodevelopmental factors^2,6^. Thus, the current “dopamine hypothesis” of schizophrenia reflects a more comprehensive imbalance between increased in the nigrostriatal pathway and deficit in mesocortical/mesolimbic pathways^6,7^.

Based on this theoretical framework, researchers have expanded their investigations to encompass the entire dopaminergic system, where physiological functions depend on the dynamic equilibrium and coordinated regulation of dopamine synthesis, receptor availability, and dopamine transporter (DAT) functionality^8^. Dopamine synthesis begins with the catalytic actions of tyrosine hydroxylase (TH) and aromatic L-amino acid decarboxylase, directly influencing the storage and release of dopamine within neurons^9^. Dopamine receptors, categorized into D_1_-like and D_2_-like families, modulate cognitive, reward, and motor functions through Gs and Gi protein-mediated signaling pathways^10^. DAT regulates the reuptake of synaptic dopamine via a sodium-dependent mechanism, thereby maintaining extracellular dopamine concentrations^11^. These components form a “synthesis-release-reuptake-signaling” loop. In pathological states like Parkinson’s disease and Schizophrenia, imbalances in any part of this loop can lead to widespread neural dysfunction through cascading effects^12,13^.

The dopamine hypothesis was originally supported by post-mortem brain tissue studies^14–16^ and cerebrospinal fluid (CSF) dopamine metabolites^17^. However, post-mortem studies do not account for the active processes of dopamine function, such as dopamine synthesis and release. Cerebrospinal fluid studies also fail to localize the region-specific dopamine dysfunction^18,19^. The rapid development of molecular imaging technology has provided important evidence for schizophrenia^20^, including positron emission tomography (PET) and single photon emission tomography (SPECT). The commercial development of SPECT predates PET^21^, but its image quality—such as spatial resolution, contrast-to-noise ratio, and biomarker specificity—is inferior to PET, resulting in less effective pathophysiological localization. The discovery of various PET tracers has deepened our understanding of the function and dysfunction of brain dopamine subsystems that assess Dopamine synthesis capacity (DSC), Dopamine transporter availability (DAT) availability, and postsynaptic dopamine receptor availability, respectively^22^. For example, D_1_ availability is mainly measured by [^11^C] NNC 112, [^11^C] SCH 23390; D_2/3_ is marked by [^11^C] FLB 457, [^18^F] fallypride and [^11^C] raclopride; DAT availability is evaluated by [^11^C] PE2I and [^18^F] CFT; and DSC is represented by L-[β-^11^C] DOPA and 6-[^18^F]-Fluorodopa.

PET tracer is used to estimate the average activity of receptor density or enzyme processes in specific brain regions, providing important evidence for revealing the “dopamine hypothesis” of schizophrenia^23^. However, previous reports on the dysfunction of the dopamine system in schizophrenia patients have great heterogeneity. For example, studies have reported either increased^24^, decreased^25^, or no abnormalities^25^ in striatal D_2_ receptor availability in schizophrenia; for D_1_ receptors, although some literature supports the PFC dopamine deficiency hypothesis^26^, there were also PET studies do not support this finding^27,28^; in terms of dopamine synthesis, both high^29^ and low capacity^30^ were reported in the same striatum. Given the high cost and diverse markers of PET, it is difficult to design a large-sample prospective study to elucidate this issue; alternatively, meta-analysis is a candidate to resolve the possible factors and hierarchical structure leading to the reported heterogeneity by summarizing existing research results. However, most meta-studies on PET dopamine dysfunction of schizophrenia focus only on the average effect of a single dimension, such as a specific dopamine subsystem, brain region, or clinical conditions^31–39^; only a few meta-analyses have focused on two or more factors^40–42^, and among them only one 2014 study examined the dysfunction distributions of different dopamine subsystems across extrastriatal cortical and subcortical regions^42^, but it did not fully elaborate on the antipsychotic effect because of limited number of reports at that time.

In this meta-analytic study, we hypothesized that dopamine dysfunctions of schizophrenia are subsystem-dependent and area-dependent. This study aimed: (1) to categorize dopamine system research into three subsystems—presynaptic synthesis, intrasynaptic transport, and postsynaptic binding—and then use meta-analysis to synthesize previous PET studies of schizophrenia across these dopamine subsystems, systematically revealing the heterogeneity of dopamine dysfunction in different brain regions of schizophrenia patients; (2) To explore potential contributing factors to this heterogeneity, such as antipsychotics, age, sex, publication year, illness duration of patients, and severity of symptoms.

## Results

**Fig. 1:**
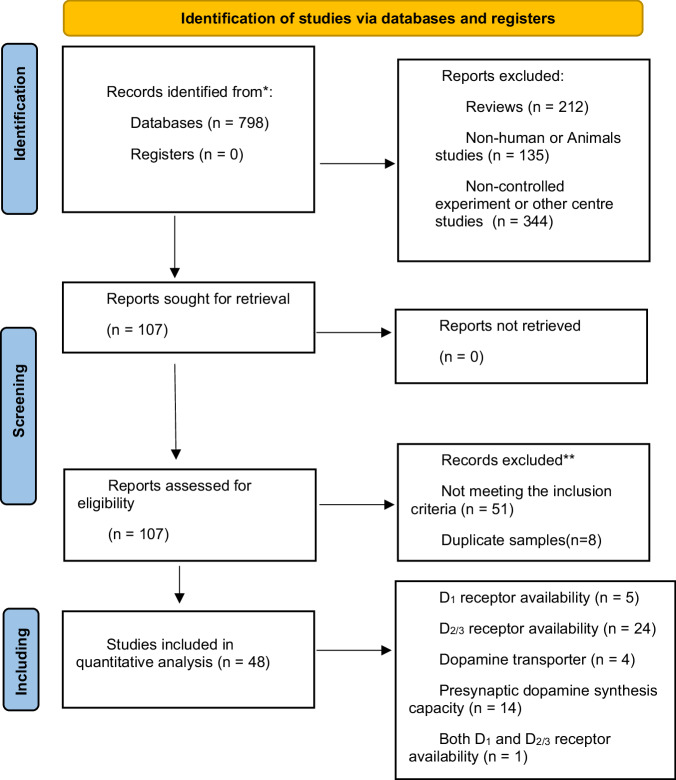
Flowchart for determining screening criteria.

After initially identifying 797 articles, 749 were excluded with the pipeline described in Fig. 1, leaving 48 PET dopamine case-control studies involving 692 patients and 730 controls for meta-analyses, including 24 studies on D_2/3_ receptors availability, 14 studies on DSC, 5 studies on D_1_ receptors availability, 4 on DAT availability, and 1 study include both D_1_ and D_2/3_ receptor availability, the specific studies included in the meta-analysis can be found in Supplementary Table 2. The detailed characteristics of the included studies are shown in Table 1 and Supplementary Table 3. The statistical findings of all meta-analyses are shown in Table 2, and all meta-regression results are shown in Table 3. Through full-text review, we identified and excluded seven studies with duplicate samples from the same research group to ensure data independence.^27,43–48^.

**Table 1:** Included schizophrenia PET studies for meta-analyses.

Study^a^	Drug history	Sex(M/F)		Age (mean ± sd)		PET Tracer	Dopamine markers	Outcome measure
		SCZ	HC	SCZ	HC			
Wong_1986_Science	DN	8/2	9/2	31.2 ± 3.6	24.3 ± 2	[^11^C] NMSP	D_2_	Bmax
	DF	5/0	9/2	26.8 ± 2.6	24.3 ± 2	[^11^C] NMSP	D_2_	Bmax
Farde_1990_Arch Gen Psychiatry	DN	10/8	10/10	24.2 ± 3.3	27.5 ± 4.9	[^11^C] Raclopride	D_2_	Bmax
Tune_1993_Psychiatry Res	DN	13/5	13/4	32.3 ± 8.5	39 ± 5.9	[^11^C] NMSP	D_2_	Bmax
	DF	4/3	13/4	41.6 ± 28.7	39 ± 5.9	[^11^C] NMSP	D_2_	Bmax
Hietala_1994_Arch Gen Psychiatry	DN	9/4	6/4	26.8 ± 7.3	25.2 ± 6.8	[^11^C] Raclopride	D_2_	Bmax
Nordstrom_1995_Psychiatry Res	DO	5/2	7/0	28.4 ± 5.7	27.7 ± 6.8	[^11^C] NMSP	D_2_	Bmax
Okubo_1997_Nature	DN	10/0	12/0	26.1 ± 3.8	27.7 ± 5.6	[^11^C] NMSP	D_2_	k_3_
	DN	10/0	6/0	26.1 ± 3.8	27.7 ± 5.6	[^11^C] SCH 23390	D_1_	k_3_/k_4_
	DF	7/0	12/0	29.2 ± 8.1	27.7 ± 5.6	[^11^C] NMSP	D_2_	k_3_
	DF	7/0	6/0	29.2 ± 8.1	27.7 ± 5.6	[^11^C] SCH 23390	D_1_	k_3_/k_4_
Breier_1997_Proc Natl Acad Sci USA	DN/DF	8/3	9/3	32.4 ± 9.9	29.2 ± 9	[^11^C] Raclopride	D_2/3_	Br
Suhara_2002_Arch Gen Psychiatry	DN	11/0	18/0	28.1 ± 7.9	27.3 ± 6.2	[^11^C] FLB 457	D_2_	BP_ND_
Talvik_2003_Int J Neuropsychopharmacol	DN	3/6	4/4	36 ± 12	31 ± 12	[^11^C] FLB 457	D_2_	BP
Yasuno_2004_Am J Psychiatry	DN	10/0	19/0	29.5 ± 7.8	29.6 ± 7.5	[^11^C] FLB 457	D_2_	BP
Grunder_2006_Neuropsychopharmacology	DO	10/5	7/0	36 ± 7.9	32 ± 6.9	[^18^F] Fallypride	D_2/3_	BP
Buchsbaum_2006_Schizophr Res	DN	10/5	9/6	28.5 ± 8.9	27.4 ± 7.9	[^18^F] Fallypride	D_2/3_	BP
Talvik_2006_Psychiatry Res	DN	9/9	13/4	28.8 ± 10.6	NA	[^11^C] Raclopride	D_2/3_	BP_ND_
Guerrero_2009_Arch Gen Psychiatry	DO	11/9	17/6	37.6 ± 7.3	34.6 ± 8.3	[^11^C]-( + )-PHNO	D_2/3_	BP_ND_
Guerrero(2)_2009_Neuropsychopharmacology	DF	9/4	9/4	25.9 ± 5.9	26.9 ± 6.4	[^11^C]-( + )-PHNO	D_2/3_	BP_ND_
Kessler_2009_Biol Psychiatry	DN/DF	6/5	5/6	30.5 ± 8	31.6 ± 9.2	[^18^F] Fallypride	D_2_	BP_ND_
Kegeles_2010_Biol Psychiatry	DN/DF	14/7	17/5	31 ± 12	26 ± 6	[^18^F] Fallypride	D_2_	BP_ND_
Kegeles(2)_2010_Arch Gen Psychiatry	DF	13/5	13/5	29 ± 8	29 ± 7	[^11^C] Raclopride	D_2_	BP_ND_
Slifstein_2015_JAMA Psychiatry	DF	10/10	11/10	33.1 ± 10.2	32.6 ± 8.1	[^11^C] FLB 457	D_2/3_	BP
Nakajima_2015_Schizophr Res	DN	3/1	5/5	66.5 ± 13	63 ± 9.2	[^11^C] Raclopride	D_2/3_	BP_ND_
	DF	2/4	5/5	66.3 ± 11.7	63 ± 9.2	[^11^C] Raclopride	D_2/3_	BP_ND_
Joo_2018_Eur Arch Psychiatry Clin Neurosci	DO	6/10	8/9	36.9 ± 11.4	32.3 ± 9.5	[^18^F] Fallypride	D_2/3_	BP_ND_
Frankle_2018_Biol Psychiatry	DF	10/4	10/4	24.5 ± 4.6	25.6 ± 4.1	[^11^C] NPA	D_2/3_	BP_ND_
Veselinovic_2018_Psychopharmacology(Berl)	DN	4/2	7/4	27.8 ± 11.3	30.6 ± 9.6	[^18^F] Fallypride	D_2/3_	BP_ND_
	DF	6/3	7/4	29.4 ± 8.2	30.6 ± 9.6	[^18^F] Fallypride	D_2/3_	BP_ND_
Schifani_2018_Brain	DN	8/6	7/5	28.3 ± 6.1	26 ± 6.5	[^11^C] FLB 457	D_2/3_	BP_ND_
Plavén-Sigray_2022_Mol Psychiatry	DN	11/8	11/8	29.3 ± 6.3	29.2 ± 5.9	[^11^C] FLB 457	D_2_	BP_ND_
Hietala_1995_Lancet	DN	4/3	6/2	26 ± 7	27 ± 7	[^18^F] DOPA	DSC	Ki
Dao-Castellana_1997_Schizophr Res	DN/DF	6/0	7/0	26 ± 9	25 ± 5	[^18^F] DOPA	DSC	Ki
Lindstrom_1999_Biol Psychiatry	DN	10/2	8/2	31.1 ± 8.6	NA	L-[β-^11^C] DOPA	DSC	Ki
Hietala_1999_Schizophr Res	DN	4/6	8/5	29.6 ± 8.8	30.4 ± 9.4	[^18^F] FDOPA	DSC	Ki
Elkashef_2000_Psychiatry Res	DF	7/2	8/5	33.3 ± 7.9	34.7 ± 10.8	[^18^F] DOPA	DSC	Ur
	DO	8/2	8/5	39.3 ± 8.7	34.7 ± 10.8	[^18^F] DOPA	DSC	Ur
McGowan_2004_Arch Gen Psychiatry	DO	16/0	12/0	37.3 ± 10.8	38.3 ± 7.1	[^18^F] DOPA	DSC	Ki
Kumakura_2007_J Neurosci	DF	8/0	15/0	37.3 ± 6.3	37.3 ± 6.4	[^18^F] DOPA	DSC	Ki
Nozaki_2009_Schizophr Res	DN/DF	10/8	10/10	35.6 ± 7.4	35.1 ± 9.5	L-[β-^11^C] DOPA	DSC	Ki
Howes_2009_Arch Gen Psychiatry	DF	5/2	8/4	36 ± 14.7	24.3 ± 4.6	[^18^F] DOPA	DSC	Ki
Demjaha_2012_Am J Psychiatry	DO	5/7	5/7	45.7 ± 9.8	44.2 ± 8.9	[^18^F] DOPA	DSC	Ki
	DO	6/6	5/7	44 ± 11.9	44.2 ± 8.9	[^18^F] DOPA	DSC	Ki
Jauhar_2017_JAMA Psychiatry	DN	14/2	14/8	26.3 ± 4.4	24.5 ± 4.5	[^18^F] DOPA	DSC	Ki
Kim_2017_ Neuropsychopharmacology	DO	8/4	8/4	31.1 ± 9.8	30.3 ± 8.4	[^18^F] DOPA	DSC	Ki
	DO	9/3	8/4	31.3 ± 8.1	30.3 ± 8.4	[^18^F] DOPA	DSC	Ki
Jauhar_2019_Mol Psychiatry	DO	10/3	10/4	24.4 ± 3	24.29 ± 4.6	[^18^F] DOPA	DSC	Ki
	DO	12/1	10/4	26.2 ± 5.8	24.3 ± 4.6	[^18^F] DOPA	DSC	Ki
Avram_2019_Brain	DO	8/15	9/15	43 ± 11.9	38.5 ± 11.6	[^18^F] DOPA	DSC	Ki
Abi-Dargham_2002_J Neurosci	DN/DF	13/3	11/5	33 ± 12	34 ± 10	[^11^C] NNC^11^2	D_1_	BP_ND_
Karlsson_2002_Am J Psychiatry	DN	8/2	8/2	24.5 ± 2.3	26.3 ± 3.6	[^11^C] SCH 23390	D_1_	BP_ND_
Kosaka_2010_Life Sci	DO	5/1	6/6	46.5 ± 8.2	42.8 ± 8.5	[^11^C] SCH 23390	D_1_	BP_ND_
Abi-Dargham_2012_J Psychopharmacol	DF	11/2	20/4	30.6 ± 10.2	30.3 ± 9.8	[^11^C] NNC ^11^2	D_1_	BP_p_
	DN	7/5	12/12	25.4 ± 4.8	26 ± 4.7	[^11^C] NNC ^11^2	D_1_	BP_p_
Stenkrona_2019_Int J Neuropsychopharmacol	DN	11/7	17^b^	32 ± 9.8	NA	[^11^C] SCH 23390	D_1_	BP_ND_
Laakso_2000_Am J Psychiatry	DN	6/3	6/3	30.1 ± 7	29.9 ± 5.6	[^18^F] CFT	DAT	BP_ND_
Laakso(2)_2000_Schizophr Res	DO	8^b^	8^b^	37.1 ± 5.7	35.3 ± 5.7	[^18^F] CFT	DAT	BP_ND_
Arakawa_2009_J Psychiatr Res	DN/DF	6/2	10/2	36.5 ± 9.5	33.2 ± 12	[^11^C] PE2I	DAT	BP_ND_
Artiges_2017_Schizophr Bull	DF/DO	21/0	30/0	34.2 ± 10.2	30.2 ± 9.7	[^11^C] PE2I	DAT	BP_ND_

*DAT* dopamine transporter, *DN* drug-naive, *DO* drug-on, *DSC* presynaptic dopamine synthesis capacity, *HC* healthy controls, *DF* drug-free, *SCZ* schizophrenia patients.

^a^Titles of studies included in the meta-analysis in Supplementary Table 2.

^b^No sex provided, NA: No age provided.

**Table 2:** Main statistics for Meta-analyses of dopamine subsystem dysfunction in schizophrenia by different PET markers.

Marker	Region	Patients	N_SCZ_	N_HC_	Cohen’s d		Meta Z	Meta P	Q	Q-test *p*	I^2^(%)	I^2^[95%CI(%)]	Egger’s *z*	Egger’s *p*	Power	
D2/3																
	STR	Total	277	313	0.41		1.91	0.056	**159.37**	**8.25e-23**	86.19	[80.53, 90.21]	1.18	0.236	0.999	
	STR	Drug Off	219	259	0.56		**2.73**	**0.006**			82.02	[72.97, 88.05]			1	
	STR	Drug Naive	97	108	0.77		**2.16**	**0.031**			87.98	[78.58, 93.25]			1	
	STR	Drug On	58	54	−0.36		-0.50	0.616			93.90	[87.56, 97.01]			0.471	
	THA	Total	193	209	−0.38		**−2.14**	**0.032**	**46.37**	**1.23e-05**	71.96	[51.97, 83.63]	−1.09	0.274	0.967	
	THA	Drug Off	162	162	−0.18		−1.46	0.144			37.97	[0.00, 68.02]			0.385	
	THA	Drug Naive	88	107	−0.26		−1.40	0.162			54.52	[0.00, 80.53]	0.45	0.650	0.436	
	THA	Drug on	31	24	−1.73		**−3.49**	**4.75e-04**			68.09	[0, 92.80]			1	
	LIB	Total	103	104	−0.41		−1.42	0.157	**32.37**	**1.38e-05**	81.47	[62.74, 90.78]	−0.66	0.509	0.835	
	LIB	Drug Off	72	80	−0.03		−0.16	0.877			39.87	[0.00, 77.78]			0.054	
	LIB	Drug On	31	24	−1.40		−**5.82**	**5.99e-09**			0.00	[0.00, 0.00]			0.999	
	SN	Total	96	91	−0.29		−0.62	0.537	**43.30**	**3.21e-08**	88.45	[77.41, 94.10]	−0.54	0.590	0.505	
	SN	Drug Off	65	67	0.33		0.96	0.335			71.02	[17.27, 89.85]			0.469	
	SN	Drug On	31	24	−1.51		−**5.65**	**1.62e-08**			0.00	[0.00, 0.00]			1	
	TMC	Total	118	126	−0.43		−1.83	0.067	**26.24**	**9.54e-04**	69.52	[39.19, 84.72]	0.07	0.941	0.917	
	TMC	Drug Off	87	102	−0.16		1.09	0.274			1.14	[0.00, 71.14]			0.193	
	TMC	Drug On	31	24	−1.50		**−3.29**	**0.001**			63.08	[0.00, 91.52]			1	
	PFC	Total	54	59	0.07		0.27	0.784	6.20	0.102	51.59	[0.00, 83.00]	0.01	0.988	0.066	
DSC																
	STR	Total	214	245	0.21		−1.30	0.193	**70.47**	**1.79e-08**	75.88	[61.98, 84.69]	1.01	0.311	0.610	
	STR	Drug Off	93	120	0.38		**2.31**	**0.021**			46.30	[34.08, 89.39]			0.782	
	STR	Drug native	45	53	0.74		**4.32**	**1.58e-05**			0	[0.00, 84.69]			0.951	
	STR	Drug On	121	125	0.06		0.20	0.839			83.76	[34.08, 89.39]			0.076	
	LIB	Total	57	71	<−0.01		−0.002	0.989	5.03	0.284	20.51	[0.00, 66.17]	−0.33	0.737	0.050	
	LIB	Drug Off	47	58	0.01		0.05	0.962			39.50	[34.08, 89.39]			0.050	
	PFC	Total	49	56	−0.23		−0.74	0.458	**7.88**	**0.048**	61.95	[0.00, 87.23]			0.214	
	TMC	Total	49	56	<−0.01		−0.03	0.976	1.61	0.657	0.00	[0.00, 84.69]			0.050	
D1																
	LIB	Total	66	92	−0.41		−1.41	0.158	**16.70**	**5.11e-03**	70.06	[29.91, 87.21]	**−3.63**	**3.00e-04**	0.714	
	LIB	Drug Off	60	80	−0.20		−0.82	0.413			55.30	[0.00, 83.49]			0.214	
	PFC	Total	91	140	−0.46		−1.10	0.269	**60.12**	**1.43e-10**	88.36	[79.35, 93.43]	**−3.13**	**0.0017**	0.925	
	PFC	Drug Off	85	128	−0.14		−0.45	0.646			82.33	[64.78, 91.14]			0.169	
	STR	Total	103	140	−0.30		−1.17	0.243	**25.59**	**5.96e-04**	72.64	[44.02, 86.63]	**−3.46**	**5.00e-04**	0.634	
	STR	Drug Off	85	128	−0.05		−0.33	0.741			20.91	[0.00, 64.27]			0.065	
	TMC	Total	66	92	−0.64		1.38	0.167	**35.71**	**6.83e-07**	86.31	[72.53, 93.25]	**−3.16**	**0.002**	0.976	
	TMC	Drug Off	60	80	−0.26		−0.79	0.427			73.55	[34.08, 89.39]			0.327	
DAT																
	STR	Total	46	59	0.34		0.58	0.564	**28.57**	**2.75e-06**	89.50	[75.92, 95.42]			0.402	

*DAT* dopamine transporter, *DSC* dopamine synthesis capacity, *LIB* limbic cortex, *PFC* prefrontal cortex, *SN* Substantia nigra, *STR* striatum, *THA* thalamus, *TMC* temporal cortex. BOLD represent statistical significance.

**Table 3:** Meta-regressions statistics of dopamine subsystem dysfunction in schizophrenia by different PET markers.

Marker	Region	Stats type	Effect β	95%CI	*z*	*p*
D2/3						
	STR	Age Effect	−0.01	[−0.05, 0.03]	−0.59	0.554
	STR	Year Effect	−0.05	[−0.09, −0.02]	**−2.83**	**0.005**
	STR	Sex Effect	4.06	[1.20, 6.92]	**2.78**	**0.005**
	STR	Duration Effect	−0.01	[−0.07, 0.04]	−0.50	0.615
	STR	Severity Effect	0.02	[−0.002, 0.05]	1.80	0.071
	THA	Age Effect	−0.09	[−0.20, 0.01]	−1.81	0.071
	THA	Year Effect	0.02	[−0.04, 0.07]	0.51	0.607
	THA	Sex Effect	−0.45	[−2.42, 1.51]	−0.45	0.652
	THA	Duration Effect	−0.01	[−0.11, 0.08]	−0.31	0.754
	THA	Severity Effect	0.01	[−0.01, 0.02]	0.83	0.406
	LIB	Age Effect	−0.07	[−0.27, 0.12]	−0.72	0.470
	LIB	Year Effect	−0.02	[−0.13, 0.10]	−0.3	0.764
	LIB	Sex Effect	−0.94	[−3.92, 2.04]	−0.62	0.536
	LIB	Duration Effect	0.03	[−0.19, 0.25]	0.26	0.794
	LIB	Severity Effect	0.03	[−0.04, 0.90]	0.83	0.407
	SN	Age Effect	−0.16	[−0.39, 0.07]	−1.37	0.171
	SN	Year Effect	−0.02	[−0.27, 0.23]	−0.17	0.867
	SN	Sex Effect	0.47	[−8.45, 9.39]	0.10	0.918
	SN	Severity Effect	0.001	[−0.12, 0.12]	−0.02	0.984
	TMC	Age Effect	−0.10	[−0.23, 0.04]	−1.43	0.152
	TMC	Year Effect	0.02	[−0.06, 0.10]	0.55	0.582
	TMC	Sex Effect	−0.40	[−3.02, 2.22]	−0.30	0.765
	TMC	Duration Effect	−0.002	[−0.12, 0.12]	−0.02	0.975
	TMC	Severity Effect	0.03	[−0.01, 0.06]	1.53	0.126
DSC						
	STR	Age Effect	−0.02	[−0.07, 0.03]	−0.85	0.397
	STR	Year Effect	−0.02	[−0.06, 0.01]	−1.21	0.227
	STR	Sex Effect	0.28	[−1.38, 1.93]	0.33	0.744
	STR	Duration Effect	−0.04	[−0.13, 0.05]	−0.95	0.344
	STR	Severity Effect	0.01	[−0.01, 0.03]	1.12	0.309
	LIB	Age Effect	−0.09	[−0.22, 0.03]	−1.52	0.129
	LIB	Year Effect	−0.04	[−0.13, 0.04]	−0.96	0.336
	LIB	Sex Effect	−1.14	[−3.66, 1.38]	−0.89	0.374
	LIB	Duration Effect	0.002	[−0.06, 0.06]	−0.07	0.945
D1						
	LIB	Age Effect	−0.06	[−0.15, 0.03]	1.33	0.183
	LIB	Year Effect	0.003	[−0.08, 0.09]	0.08	0.936
	LIB	Duration Effect	−0.08	[−0.23, 0.07]	−1.03	0.303
	LIB	Severity Effect	−0.07	[−0.14, 0.01]	−1.71	0.088
	PFC	Age Effect	−0.12	[0.22, −0.01]	**−2.23**	**0.026**
	PFC	Year Effect	0.02	[−0.10, 0.13]	0.29	0.770
	PFC	Duration Effect	−0.16	[−0.38, 0.07]	−1.35	0.177
	STR	Age Effect	−0.1	[−0.16, −0.03]	**−3.06**	**0.002**
	STR	Year Effect	−0.004	[−0.08, 0.07]	−0.1	0.921
	STR	Duration Effect	−0.16	[−0.25, −0.08]	**−3.74**	**0.0002**
	STR	Severity Effect	−0.06	[−3.36, −1.09]	**−4.20**	**0.00003**
	TMC	Age Effect	−0.11	[−0.23, 0.01]	−1.84	0.065
	TMC	Year Effect	−0.04	[−0.16, 0.09]	−0.61	0.545
	TMC	Duration Effect	−0.18	[−0.37, 0.02]	−1.74	0.08
	TMC	Severity Effect	−0.13	[−0.25, −0.01]	**−2.05**	**0.040**

*DSC* dopamine synthesis capacity, *LIB* limbic cortex, *PFC* prefrontal cortex, *SN* Substantia nigra, *STR* striatum, *TMC* temporal cortex. BOLD represent statistical significance.

### D_2/3_ receptor availability

The meta-analyses on D_2/3_ receptor availability included 375 patients and 364 controls from 25 studies, among which 5 studies separately reported DF and DN results, resulting in 30 summary statistics including 14 DN, 9 DF, 3 DN/DF, and 4 DO (Table 1).

#### Striatum

**Fig. 2:**
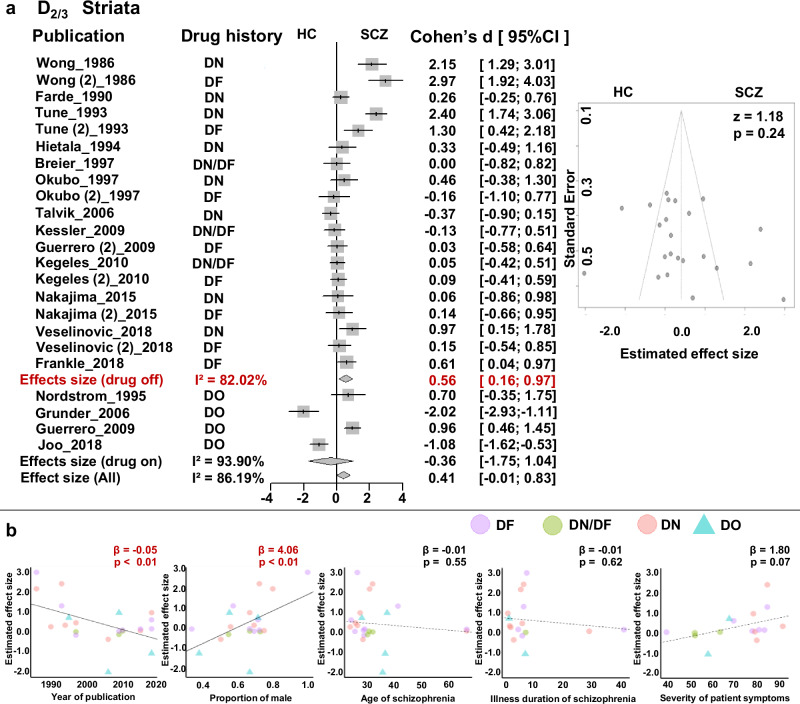
Meta-analysis of the Striatum D_2/3_ receptor availability. **a** Forrest plot and Funnel plot; **b** Meta-regression with publication year, proportion of male patients, and patients’ age. DN drug-naive, DF drug-free, DO drug-on, HC healthy control, SCZ=schizophrenia.

Meta-analysis based on all studies did not show statistical abnormality in striatum D_2/3_ receptor availability (*d* = 0.41, 95%CI = [−0.01, 0.83]) and demonstrated high heterogeneity (*Q* = 159.37, *p* < 0.01; I^2^ = 86.19%, 95%CI = [80.53%, 90.21%]) but no evidence of publication bias (Egger’s *z* = 1.18, *p* = 0.236) (Fig. 2a). When separating the studies into drug-off and drug-on sub-groups, we found a moderately increased D_2/3_ availability in the striatum of only drug-off patients (Cohen’s d = 0.56, 95% CI [0.16; 0.97]), but no significant effect in the drug-on group (*d* = −0.36, 95%CI = [−1.75, 1.04]) (Fig. 2a). We further validated the findings on DN patients with moderate D_2/3_ increased in the stratum (*d* = 0.77, 95%CI = [0.07, 1.47], Supplementary Fig. 5a). When subgroup analyses were performed by tracer type, consistently significant effects were observed in early studies using [^11^C]NMSP (*d* = 1.52, 95%CI = [0.57, 2.47]) but failed using [^11^C] Raciopride and [^11^F] Fallypride (Supplementary Fig. 1). Finally, meta-regression demonstrated a negative correlation between publication year and estimated D_2/3_ effects (β = −0.05, *p* < 0.01) and a positive correlation between the proportion of males and estimated D_2/3_ effects (β = 4.06, *p* < 0.01) (Table 3 and Fig. 2b). Did not find a significant association between reported D_2/3_ receptor availability and age, illness duration and severity of symptoms (Table 3 and Fig. 2b).

#### Thalamus

**Fig. 3:**
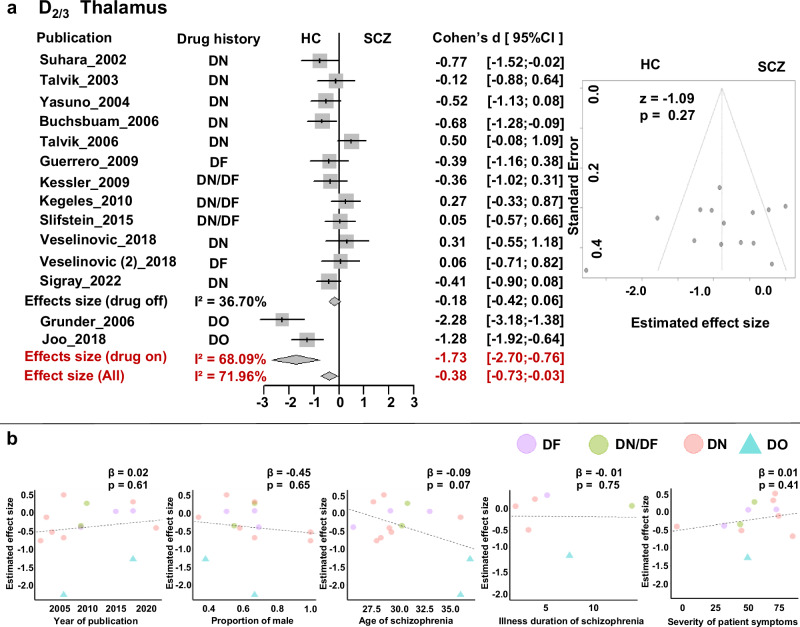
Meta-analysis of the Thalamus D_2/3_ receptor availability. **a** Forrest plot and Funnel plot; **b** Meta-regression with publication year, proportion of male patients, and patients’ age. DN drug-naïve, DF drug-free, DO drug-on, HC healthy control, SCZ schizophrenia.

In the thalamus, meta-analysis observed a small effect of decreased D_2/3_ receptor availability in patients with schizophrenia based on all studies (*d* = −0.38, 95%CI = [−0.73, −0.03]) and demonstrated medium heterogeneity (*Q* = 46.37, *p* < 0.01; I^2^ = 71.96%, 95%CI = [51.97%, 83.63], power = 0.967) but no evidence of publication bias (Egger’s *z* = −1.09, *p* = 0.274) (Fig. 3a). When separating the studies based on antipsychotic experience, we found a large effect of decreased D_2/3_ receptor availability exclusively in drug-on patients (*d* = −1.73, 95%CI = [−2.70, −0.76]) based on only two included studies^5,49^. There showed no evidence of change D_2/3_ level in drug-off (Fig. 3a) and drug naive patients (Supplementary Fig 5b). Subgroup analysis stratified by tracer type revealed consistently significant effect sizes with the use of [^11^C]FLB 457 (*d* = −0.35, 95%CI = [−0.63, −0.08]) but not significant for [^11^F] Fallypride (Supplementary Fig. 2a). Meta-regression did not find a significant association between reported thalamic D_2/3_ receptor availability and publication year, patients’ sex, and age, illness duration and severity of symptoms (Table 3 and Fig. 3b).

#### Substantia nigra, limbic, prefrontal, and temporal cortices

**Fig. 4:**
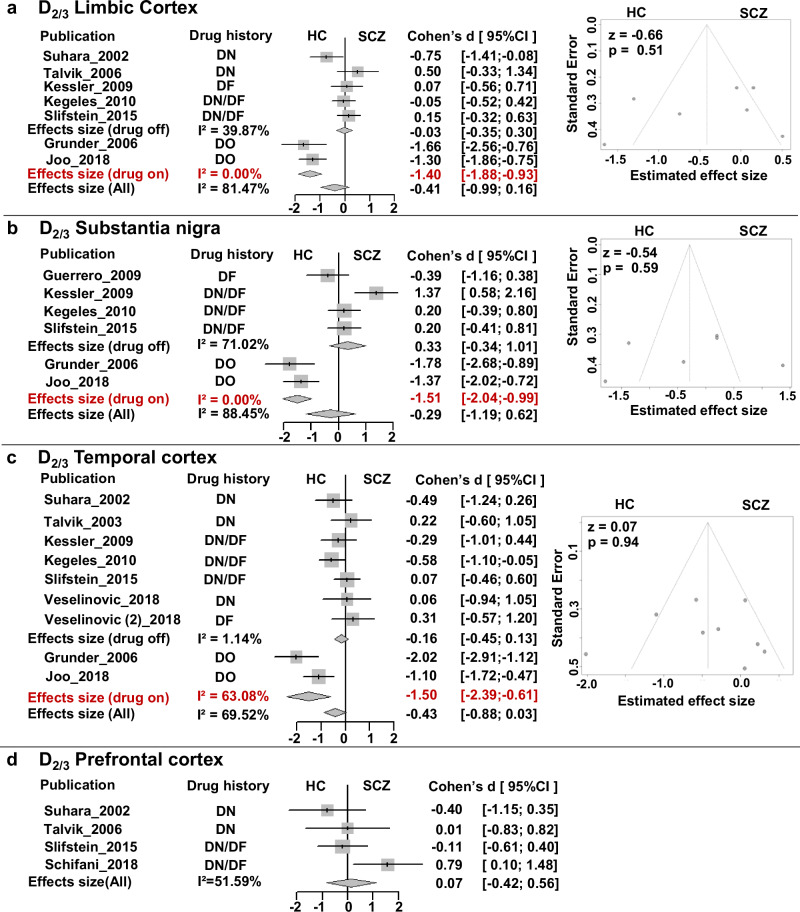
Meta-analysis of the D2/3 receptor availability outside of striatum. **a** Forrest plot and Funnel plot of Limbic cortex; **b** Forrest plot and Funnel plot of Substantia nigra; **c** Forrest plot and Funnel plot of Temporal cortex; **d** forrest plot of Prefrontal cortex. DN drug-naïve, DF drug-free, DO drug-on, HC healthy control, SCZ schizophrenia.

Based on all available studies, meta-analyses did not detect any significant abnormal D_2/3_ receptor availability in schizophrenia in either limbic cortex (Fig. 4a), substantia nigra (Fig. 4b), temporal cortex (Fig. 4c) or prefrontal cortex (Fig. 4d) and demonstrated moderate (temporal cortex I^2^ = 69.52%[39.19%, 84.72%]) to high heterogeneity (limbic cortex I^2^ = 81.47%[62.74%, 90.78%], substantia nigra I^2^ = 88.45% [77.41%, 94.10%]) but showed no evidence of publication bias in these regions across studies (*p* > 0.05). When separating the studies based on antipsychotic experience, we found a large effect of decreased D_2/3_ receptor availability exclusively in drug-on patients in limbic cortex (*d* = −1.40, 95%CI = [−1.88, −0.93]), substantia nigra (*d* = −1.51, 95%CI = [−2.04, −0.99]), and temporal cortex (*d* = −1.50, 95%CI = [−2.39, −0.61], although these findings are based on only two studies^5,49^. No significant effect was observed in drug-off patients (*p* > 0.05) in any of these regions. No significant effects were found in the subgroup analysis of tracers for the limbic cortex and temporal cortex (Supplementary Fig. 2b, 2c). Meta-regression did not find significant associations between the estimated D_2/3_ effects and publication year, patients’ sex, and age, illness duration and severity of symptoms in any of these regions (*p* > 0.05) (Table 3, Supplementary Fig. 3-4).

### Dopamine synthesis capacity (DSC)

The meta-analyses on DSC included 179 patients and 194 controls from 14 studies, resulting in 18 summary statistics, including 4 DN, 3 DF, 2 DN/DF, and 9 DO (Table 1).

#### Striatum

**Fig. 5:**
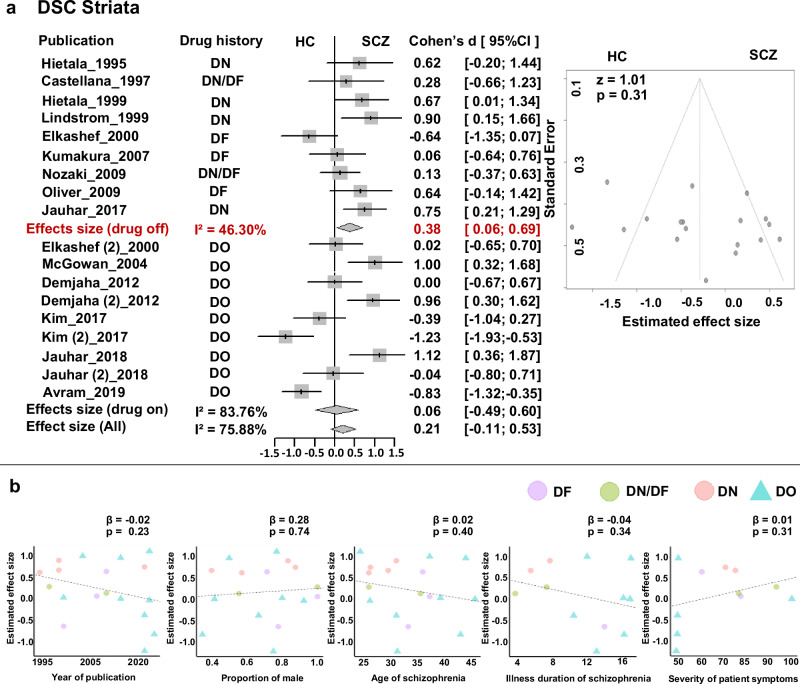
Meta-analysis of striatum dopamine synthesis capacity. **a** Forrest plot and Funnel plot; **b** Meta-regression with publication year, proportion of male patients, and patients’ age. DN drug-naive, DF drug-free, DO drug-on, HC healthy control, SCZ schizophrenia.

Meta-analysis based on all available studies did not find evidence of changed DSC in schizophrenia (*d* = 0.21, 95%CI = [-0.11, 0.53]) but demonstrated high heterogeneity (*Q* = 70.47, *p* < 0.01, I^2^ = 75.88%, 95%CI = [61.98%, 84.69%], power = 0.610) with no evidence of publication bias (Egger’s *z* = 1.01, *p* = 0.311, Fig. 5a, Table 2). When separating the studies based on antipsychotic experience, we found a small effect of increased striatal DSC exclusively in drug-off patients (*d* = 0.38, 95%CI = [0.06, 0.69], median power = 0.782), which was validated in drug-naive patients (*d* = 0.74, 95%CI = [0.40, 1.07]) (Supplementary Fig. 5c), but not in drug-on group (*d* = 0.06, 95%CI = [−0.49, 0.60]). Meta-regression analysis did not show evidence of reported DSC effects caused by publication year, patients’ sex, and age, illness duration and severity of symptoms (*p* > 0.05) (Table 3, Fig. 5b).

#### Limbic, prefrontal, and temporal cortices

**Fig. 6:**
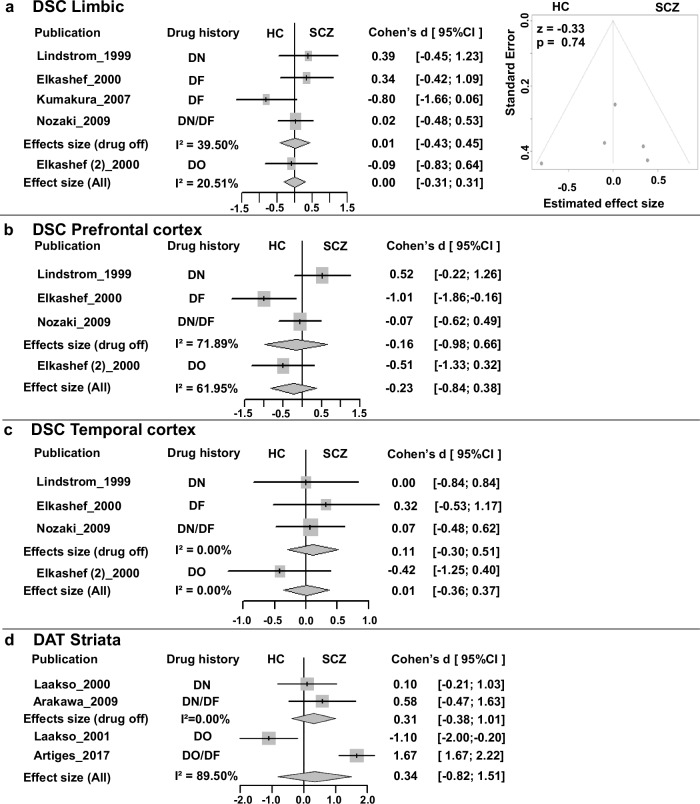
Meta-analysis of the DSC outside of the striatum and Striatum DAT. **a** Forrest plot and Funnel plot of Limbic cortex; **b** Forrest plot of Prefrontal cortex. **c** Forrest plot of Temporal cortex; **d** Forrest plot of the Striatum DAT.

Based on all available studies, meta-analyses did not detect evidence of abnormal DSC in schizophrenia in either the limbic cortex (Fig. 6a), prefrontal cortex (Fig. 6b), or temporal cortex (Fig. 6c). Sensitive analysis also demonstrated no evidence of abnormal DSC in drug-off patients. There were too few drug-on studies on DSC to permit meta-analyses, but all reported no abnormal DSC in the thalamus^50^, substantia nigra^51,52^, and limbic, prefrontal, and temporal cortices^52^. Finally, Meta-regression analysis did not show evidence of reported DSC effects caused by publication year, patients’ sex, age, illness duration and severity of symptoms (*p* > 0.05) (Table 3, Supplementary Fig. 6).

### D_1_ receptor availability

The meta-analyses on D_1_ included 92 patients and 113 controls from 6 studies, resulting in 8 summary statistics, including 4 DN, 2 DF, 1 DN/DF, and 1 DO (Table 1).

**Fig. 7:**
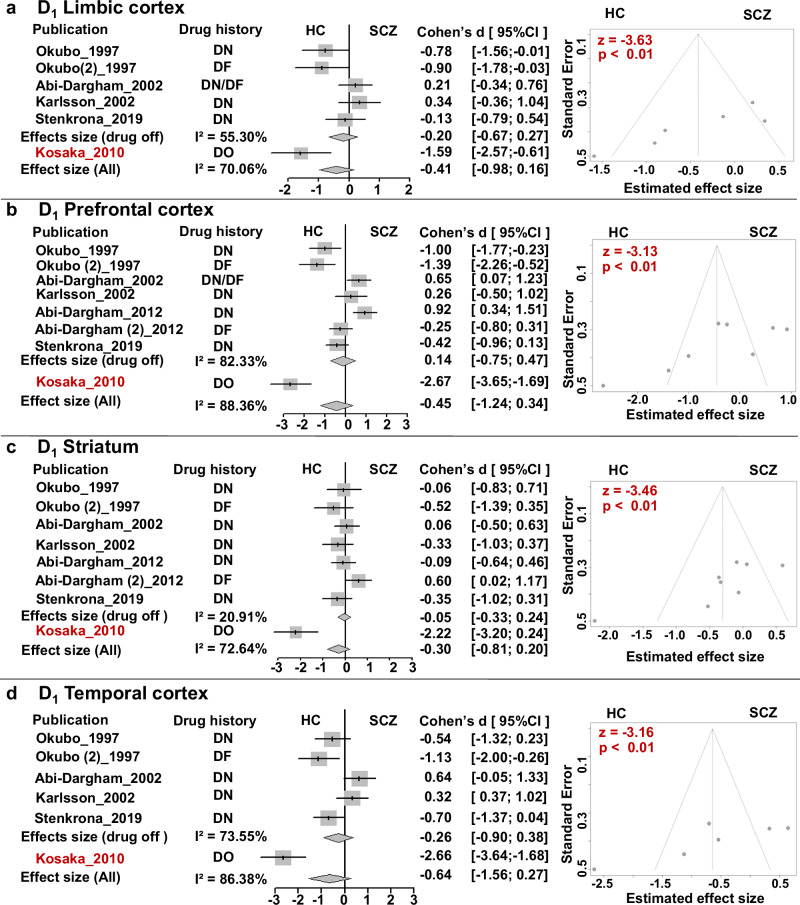
Meta-analysis of the D_1_ receptor availability. **a** Forrest plot and Funnel plot of Limbic; **b** Forrest plot and Funnel plot of Prefrontal cortex; **c** Forrest plot and Funnel plot of striatum; **d** Forrest plot and Funnel plot of Temporal cortex. DN drug-naive, DF drug-free, DO drug-on, HC healthy control, SCZ schizophrenia.

Meta-analyses based on all 6 studies did not find any evidence of abnormal D_1_ receptor availability in schizophrenia in any of the striatum, limbic cortex, prefrontal cortex, and temporal cortex (*p* > 0.05), although one DO study demonstrated reduced D_1_ availability in these regions^53^ (Fig. 7 and Table 2). Besides, the available studies demonstrated medium to high heterogeneity in these regions (I^2^: 70.06%–88.36%) and showed significant publication bias in the limbic cortex (Egger’s *z* = −3.63, *p* < 0.01), striatum (Egger’s *z* = −3.46, *p* < 0.01), prefrontal cortex (Egger’s *z* = −3.13, *p* < 0.01) and temporal cortex (Egger’s *z* = −3.16, *p* < 0.01). In only five of the drug-off studies, there also showed no evidence of abnormal D_1_ in schizophrenia in all regions (*p* > 0.05) and demonstrated medium to high heterogeneity in the limbic, prefrontal, and temporal cortices. Finally, Meta-regression showed a negative correlation between patients’ age and the estimated D_1_ effect of the prefrontal cortex (β = −0.12, *p* = 0.026) and striatum (β = −0.10, *p* < 0.01) (Table 3, Supplementary Fig. 7). Moreover, meta-regression demonstrated significantly negative correlation between striatal D_1_ receptor availability and disease duration (*z* = −3.74, *p* = 0.0002) and symptomatic severity (*z* = −4.20, *p* = 0.00003), and between temporal D_1_ receptor availability and symptoms severity (*z* = −2.05, *p* = 0.04) (Table 3 and Supplementary Fig. 8).

### Dopamine transporter availability (DAT)

The meta-analyses on DAT included 46 patients and 59 controls from 4 studies with 1 DN, 2 DF/DO, 1 DN/DF, and 1 DO (Table 1). Meta-analyses based on the 4 studies did not find any evidence of abnormal DAT (*d* = 0.34, 95%CI = [−0.82, 1.51]) but high heterogeneity across studies in the striatum (*Q* = 28.57, *p* < 0.01, I^2^ = 89.50%, 95%CI for I^2^ = [75.92%, 95.42%], power = 0.402) (Table 2, Fig. 6d). Two studies reported significantly increased DAT availability in the thalamus in either drug-off^54^ or DF/DO patients^55^. One study reported increased DAT in limbic areas and substantia nigra (*p* < 0.01)^55^.

## Discussion

Based on a meta-analysis of 48 state-of-the-art studies involving 692 patients and 730 controls, this study highlights significant heterogeneity in dopamine system dysfunction among individuals with schizophrenia in the following ways: first, there is considerable variability in both the direction (increased vs. decreased) and the involved brain regions of abnormalities across different dopamine subsystems; second, the influence of medication status varies significantly across these subsystems. Additionally, the study clarified that patients’ age, sex, disease duration and symptom severity selectively account for the reported heterogeneity of dopamine subsystem abnormalities. Finally, publication years may also bias the dopamine subsystem dysfunction.

This study highlights the increased D_2/3_ receptor availability and dopamine synthesis capacity in drug-naive and drug-free schizophrenia patients’ striatum^34^. Backing the dopamine increased hypothesis in schizophrenia’s pathophysiology^1–3^. Our results corroborate previous studies, namely that participants with greater dopamine synthesis capacity exhibit higher D_2/3_ receptor binding potential^56^. Moreover, we did not observe significant abnormal D_2/3_ receptor availability and dopamine synthesis capacity in drug-on patients. Notably, heterogeneity in D_2/3_ and DSC among drug-on patients were higher than those in drug-off patients, with both decreased^57,58^, normal^59^ and increased reported^60^. Among the four drug-on studies, one early study^59^ reported no difference in in striatal D_2/3_ receptor availability between drug-naive patients and healthy controls following immediate treatment with haloperidol (a first-generation antipsychotic). However, both groups showed a marked reduction in striatal uptake of [^11^C]NMSP. Two other studies observed reduced D_2/3_ receptor availability after long-term treatment with second-generation antipsychotics^57,58^. These findings suggest that antipsychotics, whether first- or second-generation, may relieve symptoms by reducing striatal dopamine D_2/3_ receptor availability and synthesis activity^61^. However, previous research indicates that the dopaminergic response differs between the two drug types. For example, long-term use of first-generation antipsychotics is associated with significant increases in striatal DSC and DAT expression, whereas second-generation antipsychotics have minimal effects on these presynaptic markers^62,63^. These differences likely contribute to the distinct side-effect profiles between different generation drugs^62,63^. Because there are relatively few PET studies directly comparing these drugs, more research is needed to clarify their different effects on the dopamine system.

It should be noted that one study reported increased D_3_ receptor binding in the globus pallidus (GP) using a selective D_3_ receptor affinity radiotracer [^11^C]-( + )-PHNO in patients long-term treated with second-generation antipsychotics^60^. This diversity could related to differential response of D_2_ and D_3_ receptors after antipsychotic treatment^60^. And the increased D_3_ receptor availability after treatment may be explained by antipsychotic-induced increased B_max_ by intracellular mechanisms, such as regulation of G protein signaling^64^, D_3_ mRNA Up-regulation^65^. The variability might also stem from differences in medication dosages and individual sensitivities^35^, indicating that the striatum’s functional status could be a biomarker for personalized treatment, involving not only radiotracers^32^ but also other related measures such as functional connectivity^66^. Finally, consistent with previous meta-analyses^33^, we did not find any evidence of abnormal D_1_ receptor and DAT availability in the striatum of schizophrenia, except for one D_1_ study^53^ and one DAT study^67^ that reported decreased in drug-on chronic patients. We speculated the striatum of these two dopamine subsystems may not be the key pathophysiological target for schizophrenia development, and the reduced D_1_ and DAT availability in drug-on chronic patients should be verified in the future.

While previous research suggests widespread dopamine dysfunction in schizophrenia beyond the striatum, including the mesolimbic^68^, mesocortical^69^, and thalamic-sensory pathways^70^. This study observed no abnormalities in drug-free patients. Instead, significantly reduced D_2/3_ receptor availability was observed in the thalamus, limbic areas, substantia nigra, and temporal lobe exclusively in drug-on patients, aligning with previous meta-analyses^42^. Besides, reduced D_1_ receptor availability was noted in similar regions for medicated individuals^53^. This reduction in D_2/3_ and D_1_ receptor availability outside the striatum in medicated patients might be an “overcorrection” effect of antipsychotics, potentially explaining the shift from predominantly positive symptoms in early-stage schizophrenia to more prominent negative symptoms in chronic phases^71^. These findings suggest that the mesolimbic and mesocortical hypotheses of schizophrenia may be related to antipsychotic hypodopaminergic of these pathways, which was supported by neuroimaging studies showing gray matter atrophy and functional dysconnectivity in the thalamic-sensory^72^,limbic^73^ and prefrontal subnetworks in chronic schizophrenia^74^.

The above findings reveal that antipsychotic medications exert complex effects on the dopamine subsystems across brain regions in schizophrenia. One possible reason is that mainstream antipsychotic drugs neither exclusively target a specific dopamine subsystem nor a specific brain region^75^ which may alleviate positive symptoms but potentially induce side effects such as blunted affect, reduced motivation, and social withdrawal^74^. Such findings highlight the necessity for developing targeted therapies that precisely adjust dopamine subsystems in specific brain regions, minimizing side effects and improving treatment outcomes.

We also found that the effect sizes obtained from different tracers vary significantly, demonstrating a high degree of overall heterogeneity for striatal D_2/3_ availability results (I^2^ = 85.69%). For example, early studies using [^11^C]NMSP demonstrated stronger effect but higher heterogeneity across studies, likely due to its complex and condition-dependent binding properties^59,76,77^. In contrast, studies using [^11^C]Raclopride demonstrate much lower heterogeneity and more consistent measurements, though they typically report smaller effect sizes. For instance, [^11^C]FLB 457 has exceptionally high affinity outside the striatum, particularly in the thalamus^78^, and has proven reliable in both cortical and thalamic regions^79–81^. Future studies should standardize tracer selection and continue validating tracers across different brain regions and populations to reduce heterogeneity and improve measurement consistency.

Except for drug usage, this meta-analysis also revealed that patients’ demographic and clinical conditions, including age, sex, disease duration and symptom severity, contribute selectively to the variability in dopamine abnormalities seen in schizophrenia. Notably, a higher proportion of male patients correlates with increased striatal D_2/3_ receptor availability. Previous studies have reported diverse morbidity and symptom prevalence^82^, genetic risk^83^, and brain damage patterns^84,85^ between male and female patients. Moreover, early studies demonstrated a significant interaction between dopamine receptor D_2_ gene variants and sex on cognitive control^86^, and males had generally higher D_2_ receptor affinity than women in the striatum^87^. In combination with more severe dopamine dysfunction reported in male schizophrenia^88,89^. This gender-specific observation underscores important neurobiological differences in schizophrenia. Additionally, we found older patients exhibiting severe striatal and prefrontal D_1_ receptors deficits. Age-dependent decline of dopamine D_1_ receptors had been reported in these regions^90–92^. While limited studies prevent distinguishing drug effects, this study might suggest a faster decline of striatum and prefrontal dopamine D_1_ receptors with aging in schizophrenia. Additionally, the lack of significant findings for striatal and temporal D_1_ receptor availability in our meta-analysis can be partly explained by heterogeneity in illness duration and symptom severity. Specifically, we found that longer illness duration is associated with greater reductions in striatal D_1_ availability, likely due to the accumulation of neuropathological changes and increasing disruption of the dopamine system over time—factors that can introduce confounders and bias in pooled analyses. Finally, higher symptom severity is linked to further decreases in striatal and temporal D_1_ receptor availability, as severity reflects the extent of underlying neural and neurotransmitter dysfunction, making consistent measurement and integration of results more challenging. Collectively, these sources of heterogeneity—including sex, age, illness duration, and symptom severity—limit the accuracy with which we can characterize the relationship between dopamine markers and schizophrenia. Future research should address these issues by carefully matching participants by demographic and clinical characteristics and using standardized analytic approaches. Comprehensive and stratified study designs—such as lifespan normative models^93^—will help clarify the roles of age and sex in disease progression and treatment response, leading to a more reliable understanding of schizophrenia’s pathophysiology and improving clinical care.

We observed a significant negative correlation between year of publication and the estimated effect size for striatal D_2_/D_3_ receptor availability in schizophrenia patients: studies from the 1990s frequently reported marked hyper-availability, whereas more recent research shows much smaller elevations. Notably, studies published before 1994, particularly those using the radiotracer [^11^C]NMSP, reported dramatically higher effect sizes—with Cohen’s d reaching up to 2.97 and an average effect size of 1.52 (range: 0.57–2.47). This is substantially greater than the effect sizes observed in studies using later tracers such as [^11^C]Raclopride (ES: 0.16 [–0.11, 0.43]) and [^11^F]Fallypride (ES: 0.18 [–0.19, 0.55]). Thus, radiotracer may be an important factor for publication bias. Besides, differences in statistical methods, patient demographics, and clinical characteristics may also partly contribute to the publication bias. Such bias reduces statistical power and distorts scientific conclusions, highlighting the need for reproducibility strategies, including preregistration, data sharing, and transparent reporting^94^.

This meta-analytic study faces several methodological concerns. First, the prevailing PET studies only report region-wise statistics with varying region definitions, requiring us to aggregate data into six major areas, potentially reducing spatial precision. An image-based meta-analysis with voxel-wise statistics is preferable^95^. Authors are encouraged to share unthresholded PET/SPECT maps in public repositories like Neurosynth (https://neurosynth.org/) and Neurovault (https://neurovault.org/). Second, this study focuses on case-control statistics under resting-state conditions, but dopamine receptor availability was also reported to change under specific tasks, indicating condition-dependent activity may also contribute to the reported heterogeneity of dopamine dysfunction^27^. More research on this issue is needed due to limited studies on this effect. Third, we were unable to perform stratified meta-regression separately on drug-off and drug-on patients due to the insufficient number of studies. Fourth, our power analysis demonstrates that sample size is a critical driver of heterogeneity, and thus expanding the sample is a current research priority. Future studies should be anchored in large-scale cohorts. Finally, given that patient age and medication use may confound the experimental results, along with the tracer-specific nature of PET imaging, we were unable to conduct integrated analyses across dopamine subsystems. We hope future studies with better controlled variables may uncover potential pathophysiological mechanisms underlying the disorder.

## Methods

The PRISMA (Preferred Reporting Items for Systematic Reviews and Meta-Analyses)^96^ were followed in conducting this study.

### Search and selection strategy

We conducted a comprehensive search of PubMed and Web of Science databases using the search terms [(“PET” OR “Positron emission tomography”) AND (“schizophrenia” OR “schizophrenic”) AND (“dopamine” OR “dopaminergic”)] to identify relevant studies on the research topic on January 2, 2025, without any specified time constraints for publication dates. Additionally, we manually searched the reference lists of articles in previous reviews to capture any relevant studies that may have been missed in the searches. While all included literature was in English, no language restrictions were applied. Furthermore, we applied additional screening criteria to select studies if they: (1) were original, peer-reviewed articles; (2) involved DSM/ICD-diagnosed schizophrenia patients and matched controls; (3) utilized PET imaging for comparison; (4) provided valid summary statistics to enable the derivation of Cohen’s d; and (5) For studies with overlapping samples, adjudication was performed by prioritizing those with either more comprehensive participant characteristics or larger sample sizes. Cross-examination was specifically conducted for articles published by the same research team to ensure complete independence of all included study populations.

### Data extraction and harmonization

Two reviewers (Z.Z. and X.L.) extracted data independently using a standardized form. The primary analysis was a difference in dopamine PET imaging between schizophrenia patients and healthy controls. We also obtained the following information regarding these studies: authors, journals, year of publication, drug history, gender, age, PET radiotracers, measures, and dopamine subsystems, illness duration, symptom severity. We selected the Simplified Reference Tissue Model (SRTM) as the preferred option when multiple quantification methods are available in a study.

Before conducting meta-analyses, we first converted each summary statistic result for each study into a standard effect size, Cohen’s d, and its standard error (*SE*_*d*_), regardless of the statistical measures used, such as mean (μ) ± standard deviation (SD) or T/P values plus sample size. Specifically, for result reporting the mean and SD for each group, the Cohen’s d, and its standard error (${{SE}}_{d}$) are calculated with:

1 $$d=\frac{{{\rm{\mu }}}_{{SCZ}}-{{\rm{\mu }}}_{{HC}}}{{{SD}}_{{pooled}}}$$

Where:

2 $${{SD}}_{{pooled}}=\sqrt{\frac{{{SD}}_{{SCZ}}^{2}\left({N}_{{scz}}-1\right)+{{SD}}_{{HC}}^{2}({N}_{{HC}}-1)}{{N}_{{SCZ}}+{N}_{{HC}}-2}}$$

3 $${{SE}}_{d}=\sqrt{\frac{{N}_{{SCZ}}+{N}_{{HC}}}{{N}_{{SCZ}}{N}_{{HC}}}+\frac{{d}^{2}}{2({N}_{{scz}}+{N}_{{HC}})}}$$

${N}_{{SCZ}}$ and ${N}_{{HC}}$ represent the number of schizophrenia patients and healthy controls, respectively. SD_pooled_ is a statistical measure used to combine the standard deviations from two or more groups into a single estimate of variability.

For studies only reporting t values and sample size for each group, the Cohen’s d, and ${{SE}}_{d}$ are calculated with:

4 $$d=t\times {{SE}}_{d}$$

Where:

5 $${{SE}}_{d}=\sqrt{\frac{{N}_{{SCZ}}+{N}_{{HC}}}{{N}_{{SCZ}}{N}_{{HC}}}}$$

Because the vast majority of PET studies only reported region-of-interest (ROI) statistics, and the definition of ROIs are diverse across studies, we further standardized the ROIs by aggregating all statistical results into the same framework containing six Regions: striatum, thalamus, substantia nigra, limbic region, prefrontal cortex, and temporal cortex(Supplement Table 1). Results with more than one subfield within each ROI were aggregated by calculating the grand mean of the effect size measure in a certain study using R statistical programming language version 4.2.3 with the package “Mad” (https://cran.r-project.org/web//packages/MAd/MAd.pdf).

### Statistics

For each PET tracer and each ROI, a random-effects meta-analysis model^97^ with a restricted maximum likelihood estimator (RMLE)^98^ was conducted using the aggregated Cohen’s d and ${{SE}}_{d}$ of all available studies as inputs for the “meta” package^99^. We estimated the heterogeneity using I² which describes the percentage of total variation across studies that is due to heterogeneity rather than chance:

6 $${{\rm{I}}}^{2}=100 \% \times ({\rm{Q}}-{\rm{df}})/{\rm{Q}}$$

Where Q is Cochran’s heterogeneity statistic, and df is the degrees of freedom. Negative values of I^2^ are put equal to zero so that I^2^ lies between 0% and 100%. A value of 0% indicates no observed heterogeneity and larger values show increasing heterogeneity^100^. To assess the statistical power of our meta-analysis and evaluate the robustness of all findings, we used “pwr” package in R to conduct a *post hoc* power analysis based on the pooled effect size.

Sensitivity analyses were performed when the number of studies in the subset was no fewer than five. First, we divided studies into two subgroups based on the patient’s antipsychotic history: drug-on (DO), Including patients who received antipsychotic treatment during the PET scan included in the study, and drug-off, including those who were drug-naive (DN) or drug-free (DF) who discontinued medication before the study. Second, we performed meta-analyses on studies containing only drug-naive patients. Third, funnel plots and Egger’ s tests were applied to assess the extent of publication bias^101^. Fourth, Additional subgroup analyses were performed based on the type of tracer used in the D_2_ study due to the variety of tracers employed in D_2_ research. Finally, potential factors that may explain the heterogeneity of schizophrenia dopamine subsystem dysfunctions, including publication year, patients’ gender, and patients’ age, illness duration of patients, and severity of patients symptom were evaluated using meta-regression, When analyzing symptom severity, due to heterogeneity in assessment scales across studies, we restricted our analysis to studies employing either the Brief Psychiatric Rating Scale (BPRS) or Positive and Negative Syndrome Scale (PANSS). For studies using BPRS, scores were converted to PANSS equivalents using Equipercentile linked conversion methods^102^.

## Supplementary information

Supplementary Information

## Data Availability

The data supporting the findings of this study are derived from previously published literature, as detailed in Table 1. Statistical data can be found within this paper and its supplementary information files.
