# Supplementary material for: Resolving the heterogeneity of dopamine subsystems dysfunction in schizophrenia: a PET meta-analysis
Source: Schizophrenia (Heidelb). 2025 Nov 21;11(1):139. doi: 10.1038/s41537-025-00684-0 (PMC12639120; doi:10.1038/s41537-025-00684-0)
Supplement: Supplementary file 1 — Supplementary Information [file 41537_2025_684_MOESM1_ESM.pdf]

Resolving the Heterogeneity of Dopamine Subsystems Dysfunction in Schizophrenia: a PET Meta-analysis

Zhen Zhao[1#], Xin Li[1#], Yingying Xie[1], Liyuan Lin[1], Luli Wei[1], Zhongyu Chang[1], Yun Luo[1], Haoyang Dong[1], Xue Zhang[1], Qiqi Dong[1], Chunshui Yu[1], Meng Liang[1,2], Hao Ding[1,2]*, Wen Qin[1]*

[1] Department of Radiology, Tianjin Key Lab of Functional Imaging, Tianjin Institute of Radiology and State Key Laboratory of Experimental Hematology, Tianjin Medical University General Hospital, Tianjin 300052, China

[2] School of Medical Imaging, Tianjin Medical University, Tianjin 300070, China

[#] These authors contributed equally to the work.

* Address correspondence to: Wen Qin, Email: wayne.wenqin@gmail.com; Hao Ding, Email: dhhere2005@163.com. Department of Radiology, Tianjin Key Lab of Functional Imaging, Tianjin Institute of Radiology and State Key Laboratory of Experimental Hematology, Tianjin Medical University General Hospital, Anshan Road No 154, Heping District, Tianjin 300052, China. Fax: +862260655697. Phone: +862260655697.

Supplementary Materials

Contents

Supplemental Tables:
Supplementary Table 1. According to the classification of brain regions mentioned in the literature.
Supplementary Table 2. Titles of studies included in the meta-analysis
Supplementary Table 3. Clinical features of schizophrenia for regression analysis

Supplemental Figures:

Supplementary Fig 1. Meta-analysis of subgroups stratified by tracer type in the striatum.

Supplementary Fig 2. Meta-analysis of subgroups stratified by tracer type outside of striatum.

Supplementary Fig 3. Meta-regression with publication year, proportion of male patients, and patients' age in $D_{2/3}$ receptor availability.

Supplementary Fig 4. Meta-regression with illness duration and severity of patient sympotoms in D2/3 receptor availability.

Supplementary Fig 5. Meta-analysis of the $D_{2/3}$ availability receptor and DCS in drug-naive.

Supplementary Fig 6. Meta-regression with DSC.

Supplementary Fig 7. Meta-regression with publication year and patients' age in D_1 receptor availability.

Supplementary Fig 8. Meta-regression with illness duration and severity of patient sympotoms in D1 receptor availability.

Supplementary Table 1. According to the classification of brain regions mentioned in the literature.

Region	ROIs
Limbic region	Anterior cingulate
	Amygdala
	Hippocampus
	parahippocampal gyrus
	Occipital
	Entorhinal cortex
	Insula
	subgenu of the cingulate
Prefrontal cortex	Prefrontal cortex
	DLPFC
	MPFC
	Orbitofrontal Frontal cortex
	Frontal cortex
SN	substantia nigra
Striatum	Caudate
	Putamen
	Striatum(ventral)

	Striatum(associative)
	Striatum(sensorimotor)
	Striatum(dorsal)
	Striatum(limbic)
	Globus pallidus
Thalamus	Medial Thalamus
	Posterior Thalamus
Temproal cortex	Temproal cortex
	Lateral Temproal cortex
	Medial temporal
	Uncus
	Superior Temproal cortex
	Inferior temporal gyrus

* Some ROIs involve left, right, ventral, and dorsal sides, which will not be listed in detail here

Supplementary Table 2 Titles of studies included in the meta-analysis

Study	Title
Wong_1986_Science[1]	Positron Emission Tomography Revels -Elevated D_2 Dopamine Receptors in Drug-Naive Schizophrenics
Farde_1990_Arch Gen Psychiatry[2]	D2 Dopamine Receptors in Neuroleptic-Naive Schizophrenic Patients
Tune_1993_Psychiatry Res[3]	Dopamine D_2 Receptor Density Estimates in Schizophrenia: A Positron Emission Tomography Study With ^{11}C-*N*-Methylspiperone
Hietala_1994_Arch Gen Psychiatry[4]	Striatal D_2 Dopamine Receptor Characteristics in Neuroleptic-Naive Schizophrenic Patients Studied With Positron Emission Tomography
Nordstrom_1995_Psychiatry Res[5]	No elevated D_2 dopamine receptors in neuroleptic-naïve schizophrenic patients revealed by positron emission tomography and [^{11}C]N-methylspiperone
Okubo_1997_Nature[6]	Decreased prefrontal dopamine D_1 receptors in schizophrenia revealed by PET
Breier_1997_Proc Natl Acad Sci USA[7]	Schizophrenia is associated with elevated amphetamine-induced synaptic dopamine concentrations: Evidence from a novel positron emission tomography method
Suhara_2002_Arch Gen Psychiatry[8]	Decreased Dopamine D_2 Receptor Binding in the Anterior Cingulate Cortex in Schizophrenia
Talvik_2003_Int J Neuropsychopharmacol[9]	Decreased thalamic D_2/D_3 receptor binding in drug-naive patients with schizophrenia: a PET study with [^{11}C]FLB 457
Yasuno_2004_Am J Psychiatry[10]	Low Dopamine D_2 Receptor Binding in Subregions of the Thalamus in Schizophrenia
Grunder_2006_Neuropsychopharmacology[11]	The Striatal and Extrastriatal D_2/D_3 Receptor-Binding Profile of Clozapine in Patients with Schizophrenia
Buchsbaum_2006_Schizophr Res[12]	D_2/D_3 dopamine receptor binding with [F-18]fallypride in thalamus and cortex of patients with schizophrenia

Talvik_2006_Psychiatry Res[13]	Dopamine D_2 receptor binding in drug-naïve patients with schizophrenia examined with raclopride-C11 and positron emission tomography
Guerrero_2009_Arch Gen Psychiatry[14]	The Effect of Antipsychotics on the High-Affinity State of D_2 and D_3 Receptors
Guerrero(2)_2009_Neuropsychopharmacology[15]	The Dopamine D_2 Receptors in High-Affinity State an D_3 Receptors in Schizophrenia: A Clinical [^{11}C]-(+)-PHNO PET Study
Kessler_2009_Biol Psychiatry[16]	Dopamine D_2 Receptor Levels in Striatum, Thalamus, Substantia Nigra, Limbic Regions, and Cortex in Schizophrenic Subjects
Kegeles_2010_Biol Psychiatry[17]	Striatal and Extrastriatal Dopamine D_2/D_3 Receptors in Schizophrenia Evaluated With [^{18}F]fallypride Positron Emission Tomography
Kegeles(2)_2010_Arch Gen Psychiatry[18]	Increased Synaptic Dopamine Function in Associative Regions of the Striatum in Schizophrenia
Slifstein_2015_JAMA Psychiatry[19]	Deficits in Prefrontal Cortical and Extrastriatal Dopamine Release in Schizophrenia
Nakajima_2015_Schizophr Res[20]	Dopamine $D_{2/3}$ receptor availability in the striatum of antipsychotic-free older patients with schizophrenia A[^{11}C]-raclopride PET study
Joo_2018_Eur Arch Psychiatry Clin Neurosci[21]	The relationship between excitement symptom severity and extrastriatal dopamine -$D_{2/3}$ receptor availability in patients with schizophrenia: a high-resolution PET study with - [18F]fallypride
Frankle_2018_Biol Psychiatry[22]	Amphetamine-Induced Striatal Dopamine Release Measured With an Agonist Radiotracer in Schizophrenia
Veselinovic_2018_Psychopharmacology(Berl)[23]	The role of striatal dopamine $D_{2/3}$ receptors in cognitive performance in drug-free patients with schizophrenia
Schifani_2018_Brain[24]	Cortical stress regulation is disrupted in schizophrenia but not in clinical high risk for psychosis
Sigray_2022_Mol Psychiatry[25]	Thalamic dopamine D2-receptor availability in schizophrenia: a study on antipsychotic-naive

	patients with first-episode psychosis and a meta-analysis
Hietala_1995_Lancet[26]	Presynaptic dopamine function in striatum of neuroleptic-naive schizophrenic patients
Castellana_1997_Schizophr Res[27]	Presynaptic dopaminergic function in the striatum of schizophrenic patients
Lindstrom_1999_Biol Psychiatry[28]	Increased Dopamine Synthesis Rate in Medial Prefrontal Cortex and Striatum in Schizophrenia Indicated by L-(β-^{11}C) DOPA and PET
Hietala_1999_Schizophr Res[29]	Depressive symptoms and presynaptic dopamine function in neuroleptic-naive schizophrenia
Elkashef_2000_Psychiatry Res[30]	6-^{18}F-DOPA PET study in patients with schizophrenia
McGowan_2004_Arch Gen Psychiatry[31]	Presynaptic Dopaminergic Dysfunction in Schizophrenia
Kumakura_2007_J Neurosci[32]	Elevated [^{18}F]Fluorodopamine Turnover in Brain of Patients with Schizophrenia: An [^{18}F]Fluorodopa/Positron Emission Tomography Study
Nozaki_2009_Schizophr Res[33]	Regional dopamine synthesis in patients with schizophrenia using L-[β-^{11}C]DOPA PET
Howes_2009_Arch Gen Psychiatry[34]	Elevated Striatal Dopamine Function Linked to Prodromal Signs of Schizophrenia
Demjaha_2012_Am J Psychiatry[35]	Dopamine Synthesis Capacity in Patients With Treatment-Resistant Schizophrenia
Jauhar_2017_JAMA Psychiatry[36]	A Test of the Transdiagnostic Dopamine Hypothesis of Psychosis Using Positron Emission Tomographic Imaging in Bipolar Affective Disorder and Schizophrenia
Kim_2017_Neuropsychopharmacology[37]	Presynaptic Dopamine Capacity in Patients with Treatment-Resistant Schizophrenia Taking Clozapine: An [^{18}F]DOPA PET Study
Jauhar_2019_Mol Psychiatry[38]	Determinants of treatment response in first-episode psychosis: an ^{18}F-DOPA PET study

Avram_2019_Brain[39]	Reduced striatal dopamine synthesis capacity in patients with schizophrenia during remission of positive symptoms
Abi-Dargham_2002_J Neurosci[40]	Prefrontal Dopamine D1 Receptors and Working Memory in Schizophrenia
Karlsson_2002_Am J Psychiatry[41]	PET Study of D_1 Dopamine Receptor Binding in Neuroleptic-Naive Patients With Schizophrenia
Kosaka_2010_Life Sci[42]	Decreased binding of [^{11}C]NNC112 and [^{11}C]SCH23390 in patients with chronic schizophrenia
Abi-Dargham_2012_J Psychopharmacol[43]	Increased prefrontal cortical D_1 receptors in drug naive patients with schizophrenia: a PET study with [^{11}C]NNC112
Stenkrona_2019_Int J Neuropsychopharmacol[44]	D_1-Dopamine Receptor Availability in First-Episode Neuroleptic Naive Psychosis Patients
Laakso_2000_Am J Psychiatry[45]	Striatal Dopamine Transporter Binding in Neuroleptic-Naive Patients With Schizophrenia Studied With Positron Emission Tomography
Laakso_2001_Schizophr Res[46]	Decreased striatal dopamine transporter binding in vivo in chronic schizophrenia
Arakawa_2009_J Psychiatr Res[47]	Increase in thalamic binding of [^{11}C]PE2I in patients with schizophrenia: A positron emission tomography study of dopamine transporter
Artiges_2017_Schizophr Bull[48]	Striatal and Extrastriatal Dopamine Transporter Availability in Schizophrenia and Its Clinical Correlates: A Voxel-Based and High-Resolution PET Study

Supplementary Table 3. Clinical features of schizophrenia for regression analysis

Study	Drug history	markers	illness duration(mean ± sd)	Patient status	Time of durg-off	Drug	dosage of drug-on	Rating Scale tpye	Symptom severity(mean ± sd)
Wong_1986_Science	DN	D_2	5 ± 3	chronic	Not provided	haloperidol	Not provided	Not provided	Not provided
	DF	D_2	7 ± 2		2.6 ± 2.5 mon				
Farde_1990_Arch Gen Psychiatry	DN	D_2	1.63 ± 2.14	FEP	Not provided	Not provided	Not provided	CPRS	12 ± 3.7
Tune_1993_Psychiatry Res	DN	D_2	5.95 ± 2.07	chronic	Not provided	Not provided	Not provided	BPRS	47.25 ± 5.90
	DF	D_2						BPRS	47.25 ± 5.90
Hietala_1994_Arch Gen Psychiatry	DN	D_2	1.31 ± 1.29	Not provided	Not provided	Not provided	Not provided	BPRS	51.38 ± 18.15
Nordstrom_1995_Psychiatry Res	DO	D_2	1.10 ± 1.07	FEP	Not provided	haloperidol	7.5mg/day	BPRS	37.31 ± 29.27
Okubo_1997_Nature	DN	D_2	5.5 ± 4.92	Not provided	Not provided	Not provided	Not provided	Not provided	Not provided
	DN	D_1							
	DF	D_2							
	DF	D_1							
Breier_1997_Proc Natl Acad Sci USA	DN/DF	$D_{2/3}$	6.6 ± 1.8	chronic	23.2 ± 7.2 days	Not provided	Not provided	BPRS	28.8 ± 7.2

Suhara_2002_Arch Gen Psychiatry	DN	D_2	2.1 ± 2.29	Not provided	Not provided	Not provided	Not provided	Not provided	Not provided
Talvik_2003_Int J Neuropsychopharmacol	DN	D_2	2.78 ± 1.81	Not provided	Not provided	Not provided	Not provided	PANSS	83.33 ± 21.33
Yasuno_2004_Am J Psychiatry	DN	D_2	Not provided	Not provided	Not provided	Not provided	Not provided	BPRS	29.3±8.9
Grunder_2006_Neuropsychopharmacol	DO	$D_{2/3}$	Not provided	Not provided	Not provided	clozapine	230 ± 88.2mg/day	Not provided	Not provided
Buchsbaum_2006_Schizophr Res	DN	$D_{2/3}$	3.18 ± 4.93	Not provided	Not provided	Not provided	Not provided	BPRS	52.7 ± 9.1
Talvik_2006_Psychiatry Res	DN	$D_{2/3}$	2.65 ± 1.58	Not provided	Not provided	Not provided	Not provided	PANSS	80.56 ± 19.23
Guerrero_2009_Arch Gen Psychiatry	DO	$D_{2/3}$	Not provided	Not provided	Not provided	Olanzapine (n=7) Clozapine (n=7) Risperidone (n=6)	Olanzapine 17.86 ± 7.83mg/day Clozapine 328.5 ± 149.60mg/day Risperidone 2.79 ± 1.99 mg/day	Not provided	Not provided
Guerrero(2)_2009_Neuropsychopharmacol	DF	$D_{2/3}$	Not provided	Not provided	Not provided	Not provided	Not provided	PANSS	39.9 ± 5.40
Kessler_2009_Biol Psychiatry	DN/DF	D_2	Not provided	Not provided	Not provided	Not provided	Not provided	BPRS	28.82 ± 6.71

Kegeles_2010_Biol Psychiatry	DN/DF	D_2	Not provided	Not provided	0.52 ± 1.41	Not provided	Not provided	PANSS	64 ± 15
Kegeles(2)_2010_Arch Gen Psychiatry	DF	D_2	Not provided	chronic	Not provided	Not provided	Not provided	PANSS	78.61 ± 20.63
Slifstein_2015_JAMA Psychiatry	DN/DF	$D_{2/3}$	13.2 ± 11.3	Not provided	3.2 ± 5.6	Not provided	Not provided	PANSS	59.1 ± 10.98
Nakajima_2015_Schizophr Res	DN	$D_{2/3}$	27.8 ± 22.2	chronic	Not provided	Not provided	Not provided	PANSS	79.8 ± 22.6
	DF	$D_{2/3}$	39.4 ± 15.6					PANSS	84.1 ± 33.5
Joo_2018_Eur Arch Psychiatry Clin Neurosci	DO	$D_{2/3}$	6.5 ± 3.7	Not provided	Not provided	paliperidone(n = 5) aripiprazole (n = 3) quetiapine(n = 3) olanzapine(n = 2) risperidone (n = 2) ziprasidone (n = 1)	paliperidone 3.6 ± 1.3 aripiprazole 4.7 ± 4.6 quetiapine 410.9 ± 43.2 olanzapine 5.0 ± 3.5 risperidone 1.5 ± 0.7 ziprasidone 100.0	PANSS	58.6 ± 17.8
Frankle_2018_Biol Psychiatry	DF	$D_{2/3}$	Not provided	Not provided	39.6 ± 41.4 week	Not provided	Not provided	PANSS	70.1 ± 13.9
Veselinovic_2018_Psychopharmacology(Berl)	DN	$D_{2/3}$	0.7 ± 0.4	Not provided	Not provided	Not provided	Not provided	PANSS	79 ± 9.2

	DF	$D_{2/3}$	4.1 ± 6.4		至少停药 6 个月		Not provided	PANSS	81.6 ± 13.3
Schifani_2018_Brain	DN	$D_{2/3}$	Not provided	Not provided	Not provided	Not provided	Not provided	PANSS	69.35 ± 11.50
Sigray_2022_Mol Psychiatry	DN	D_2	12.1 ± 19.5	FEP	Not provided	Not provided	Not provided	PANSS	65.6 ± 17.4
Hietala_1995_Lancet	DN	DSC	Not provided	Not provided	Not provided	Not provided	Not provided	Not provided	Not provided
Castellana_1997_Schizophr Res	DN/DF	DSC	6 ± 8	Not provided	Not provided	Not provided	Not provided	PANSS	97 ± 19.03
Lindstrom_1999_Biol Psychiatry	DN	DSC	6.40 ± 8.79	Not provided	Not provided	Not provided	Not provided	CGI	5.29 ± 0.94
Hietala_1999_Schizophr Res	DN	DSC	4.08 ± 2.72	chronic	Not provided	Not provided	Not provided	PANSS	77.6 ± 13.06
	DF	DSC	13.0 ± 1.6			Not provided	Not provided	Not provided	Not provided
Elkashef_2000_Psychiatry Res	DO	DSC	15.3 ± 1.6	chronic	42.3±7.9 days	clozapine(n = 6) haloperidol (n = 3) fluphenazine(n=1)	425±123	Not provided	Not provided
McGowan_2004_Arch Gen Psychiatry	DO	DSC	10.9 ± 7.08	Not provided	Not provided	haloperidol	663 ± 718.75	Not provided	Not provided
Kumakura_2007_J Neurosci	DF	DSC	Not provided	Not provided	Not provided	Not provided	Not provided	PANSS	80.1 ± 4.7

Nozaki_2009_Schizophr Res	DN/DF	DSC	2.20 ± 2.72	Not provided	Not provided	Not provided	Not provided	PANSS	79.2 ± 21.4
Oliver_2009_Arch Gen Psychiatry	DF	DSC	Not provided	Not provided	Not provided	Not provided	Not provided	PANSS	61.7 ± 31.0
Demjaha_2012_Am J Psychiatry	DO	DSC	16.1 ± 8.6	Not provided	Not provided	Olanzapine (n = 3) Quetiapine(n = 2) Amisulpride(n = 1) Risperidone(n = 1) Risperidone depot(n = 2) Chlorpromazine(n = 1) Zuclopenthixol decanoate(n = 1) Flupenthixol	396.1 ± 157.5	PANSS	104.3 ± 10.6

	DO	DSC	16.2 ± 10.1	Not provided	Not provided	decanoate(n = 1) Olanzapine (n = 2) Quetiapine(n = 1) Amisulpride(n = 1) Risperidone depot(n = 3) Aripiprazole(n = 1) Zuclopenthixol decanoate(n = 3) Flupenthixol decanoate(n = 1)	283.9±159.14	PANSS	50.7 ± 5.8
Jauhar_2017_JAMA Psychiatry	DN	DSC	Not provided	Not provided	Not provided	Not provided	Not provided	PANSS	72.94 ± 16.46
Kim_2017_ Neuropsychopharmacology	DO	DSC	9.28 ± 9.02	chronic	Not provided	Risperidone (5) Paliperidon	Risperidone 4.0 ± 1.5 Paliperidone	PANSS	50.3 ± 11.1

						e (3) Olanzapine (4)	8.0 ± 1.7 Olanzapine 11.9 ± 12.1		
	DO	DSC	12.06 ± 6.48		Not provided	Clozapine	282.3 ± 126.9	PANSS	49.7 ± 7.9
Jauhar_2018_Mol Psychiatry	DO	DSC	Not provided	chronic	Not provided	Not provided	Not provided	Not provided	Not provided
	DO	DSC	Not provided						
Avram_2019_Brain	DO	DSC	15.39 ± 9.67	chronic	Not provided	Amisulpride(n = 2) Aripiprazole(n = 7) Flupentixol (n = 3) Haloperidol (n = 1) Olanzapine (n = 9) Paliperidone(n = 2) Perazine(n = 1) Perphenazine(n = 1) Pipampero	540.74 ± 409.04	PANSS	50.21 ± 12.70

ne(n = 1)
Quetiapine(
n = 4)
Risperidon
e(n = 4)
Biperiden(n
= 2)
Bupropion(
n = 2)
Citalopram
(n = 2)
Duloxetine(
n = 1)
Escitalopra
m(n = 1)
Lithium(n
= 2)
Methylphe
nidate(n =
1)
Sertraline(n
= 2)
Trimiprami
ne(n = 1)
Valproate(n
= 1)

						Venlafaxine(n = 2)<br>Zolpidem(n = 1)			
Abi-Dargham_2002_J Neurosci	DN/DF	D_1	6.8 ± 7.1	Not provided	164±173 days	Not provided	Not provided	PANSS	71 ± 11.58
Karlsson_2002_Am J Psychiatry	DN	D_1	8.9±6.4	Not provided	Not provided	Not provided	Not provided	BPRS	46.3 ± 7.42
Kosaka_2010_Life Sci	DO	D_1	18.3±10.5	Not provided	Not provided	sulpiride	966.7±294.4	PANSS	93.7 ± 18.1
Abi-Dargham_2012_J Psychopharmacol	DF	D_1	Not provided	Not provided	Not provided	Not provided	Not provided	PANSS	66.88 ± 21.79
	DN	D_1					Not provided	PANSS	61.82 ± 13.14
Stenkrona_2019_Int J Neuropsychopharmacol	DN	D_1	Not provided	Not provided	Not provided	Not provided	Not provided	BPRS	41.89 ± 9.01
Laakso_2000_Am J Psychiatry	DN	DAT	Not provided	Not provided	Not provided	Not provided	Not provided	PANSS	73 ± 23
Laakso_2000_Schizophr Res	DO	DAT	Not provided	chronic	Not provided	haloperidol (n = 2)<br>zuclopenthixol(n = 3)<br>perphenazine(n = 1)<br>thioridazine (n = 1)	Not provided	PANSS	54 ± 21

						risperidone and chlorprotixene(n = 1)			
Arakawa_2009_J Psychiatr Res	DN/DF	DAT	2.68 ± 3.57	Not provided	Not provided	Not provided	Not provided	PANSS	77.8 ± 18.8
Artiges_2017_Schizophr Bull	DF/DO	DAT	13.57 ± 9.25	Not provided	Not provided	risperidone (n = 8) aripiprazole (n = 6) olanzapine(n = 2) haloperidol (n = 2) clozapine(n = 1) clopentixol (n=1)	310.95±197.54	PANSS	79.42 ± 20.42

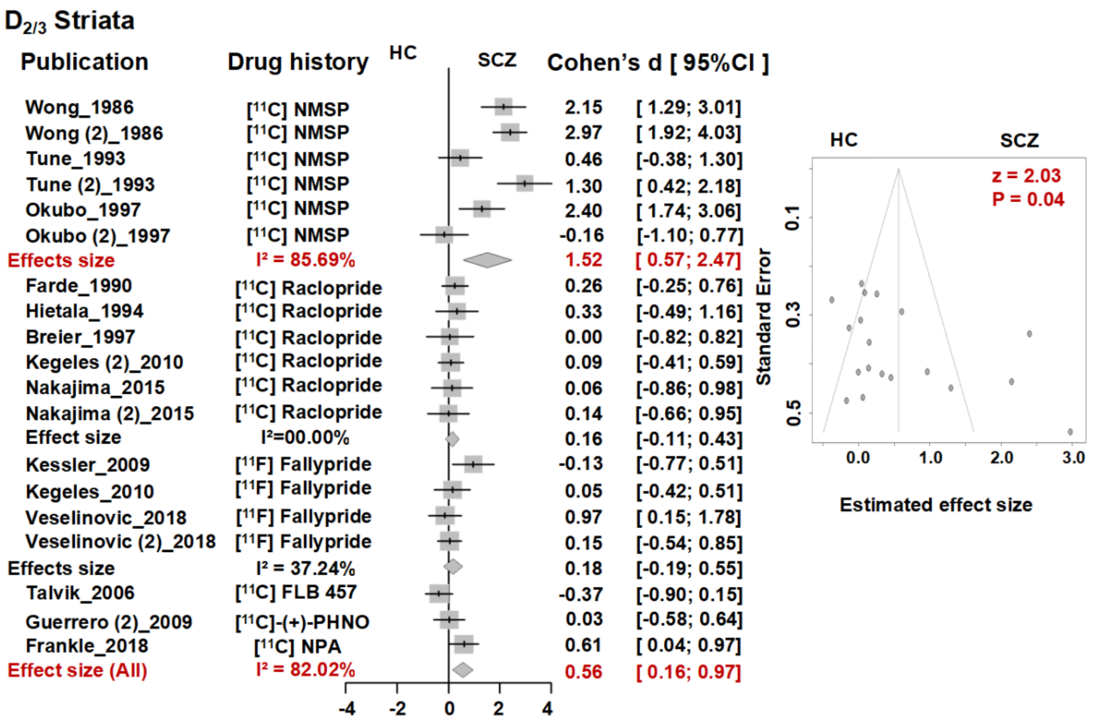

Supplementary Fig 1. Meta-analysis of subgroups stratified by tracer type in the striatum.

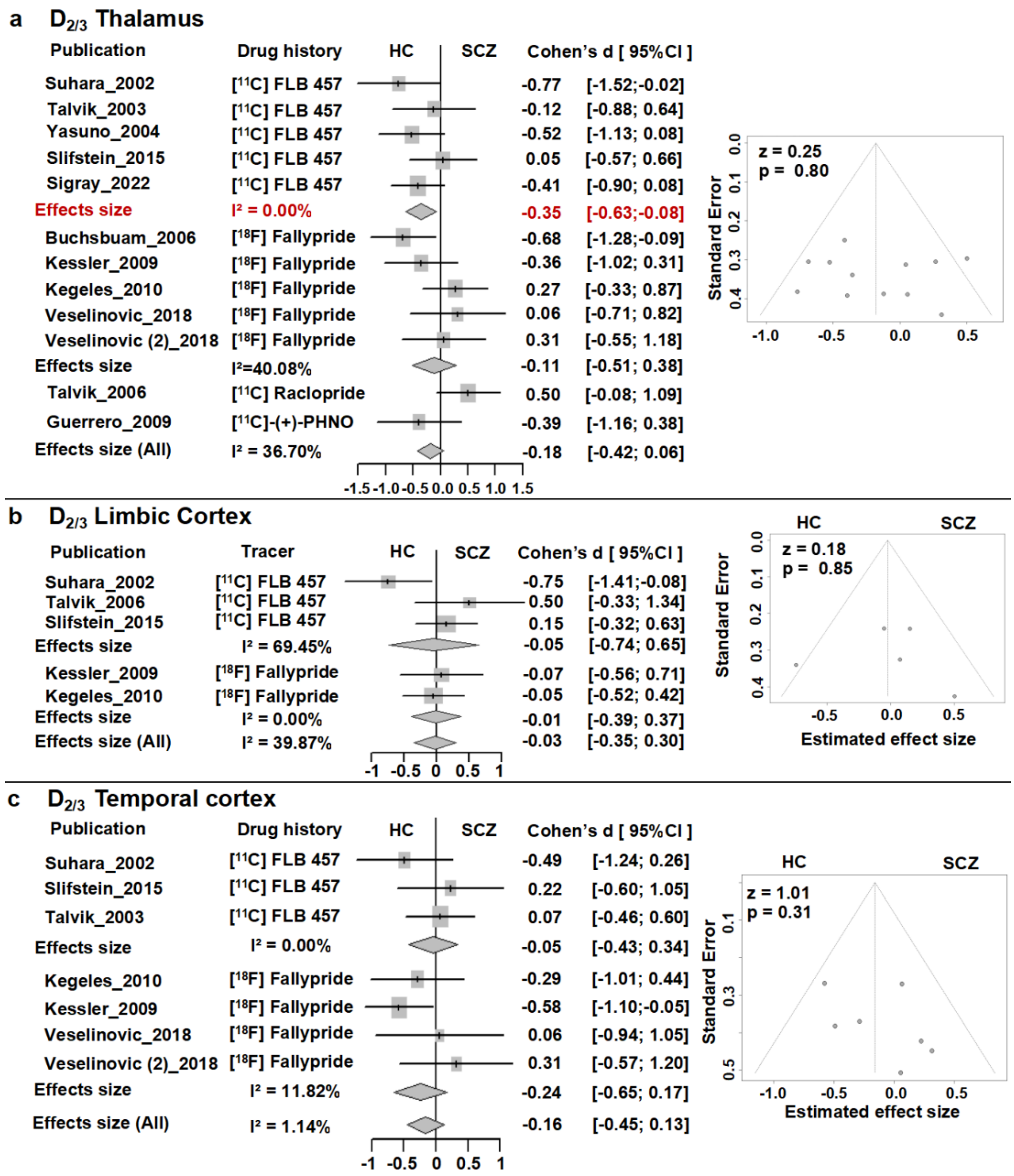

Supplementary Fig 2. Meta-analysis of subgroups stratified by tracer type outside of striatum. (a) Forrest plot and Funnel plot of Thalamus; **(b)** Forrest plot and Funnel plot of Limbic cortex; **(c)** Forrest plot and Funnel plot of Temporal cortex

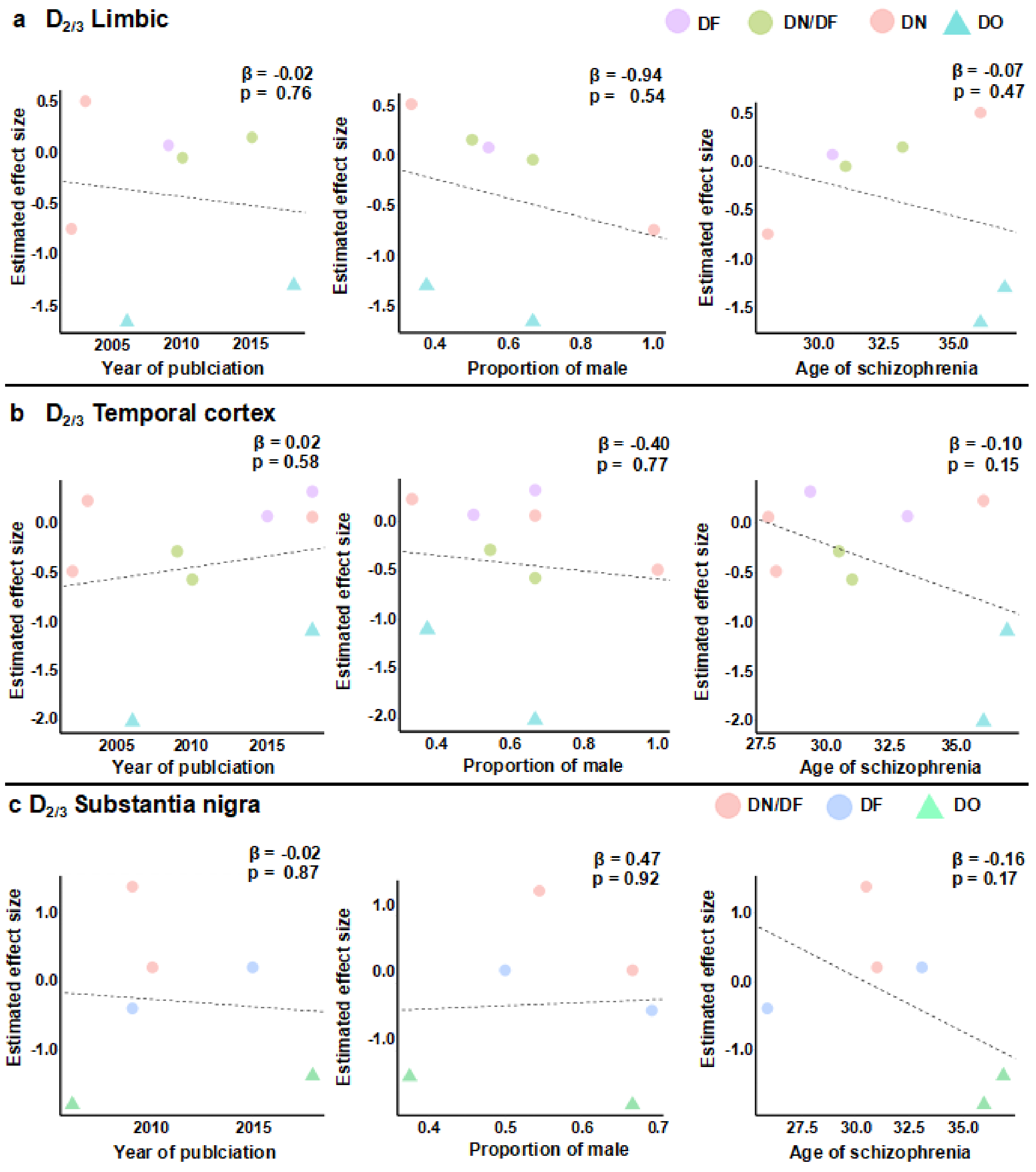

Supplementary Fig 3. Meta-regression with publication year, proportion of male patients, and patients' age in $D_{2/3}$ receptor availability. Limbic cortex **(a)**, Temporal cortex **(b)**, and Substantia nigra **(c)**.

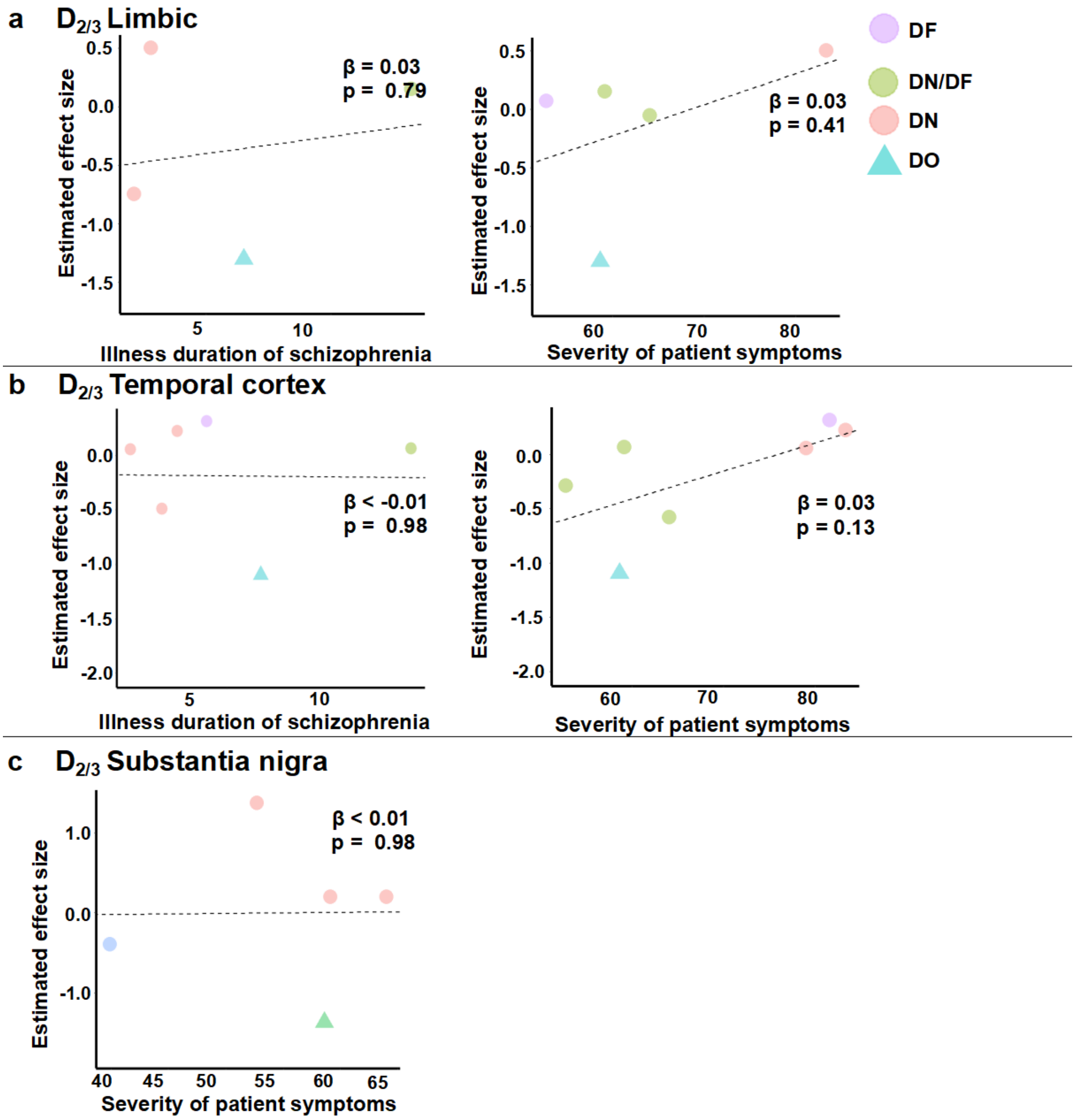

Supplementary Fig 4. Meta-regression with illness duration and severity of patient sympotoms in $D_{2/3}$ receptor availability. Limbic cortex **(a)**, Temporal cortex **(b),** and Substantia nigra **(c)**.

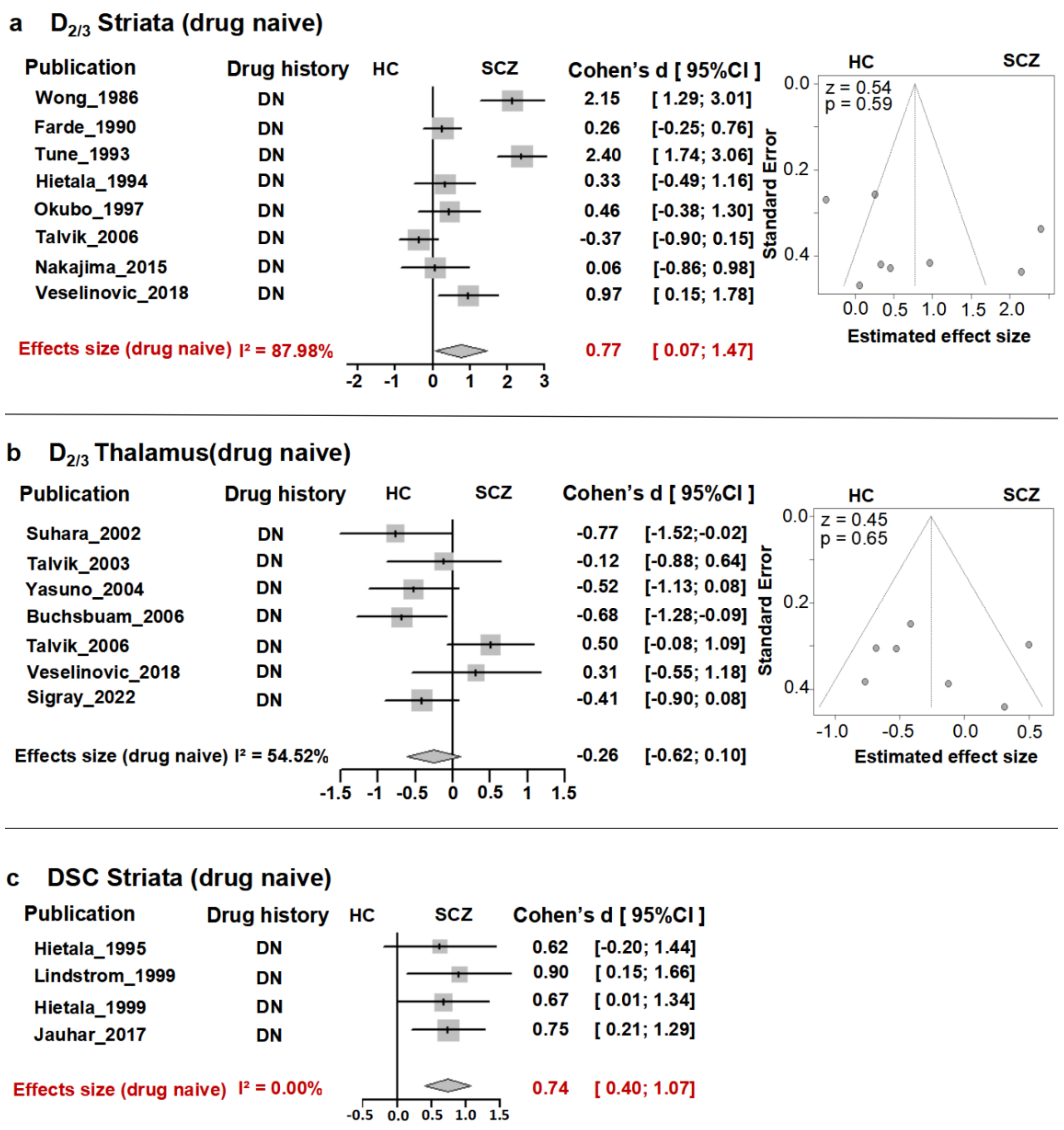

Supplementary Fig 5. Meta-analysis of the $D_{2/3}$ availability receptor and DCS in drug-naive. (a) Forrest plot and Funnel plot of Striatum D2/3 receptor; **(b)** Forrest plot and Funnel plot of Thalamus D2/3 receptor; **(c)** Forrest plot of Striatum DSC.

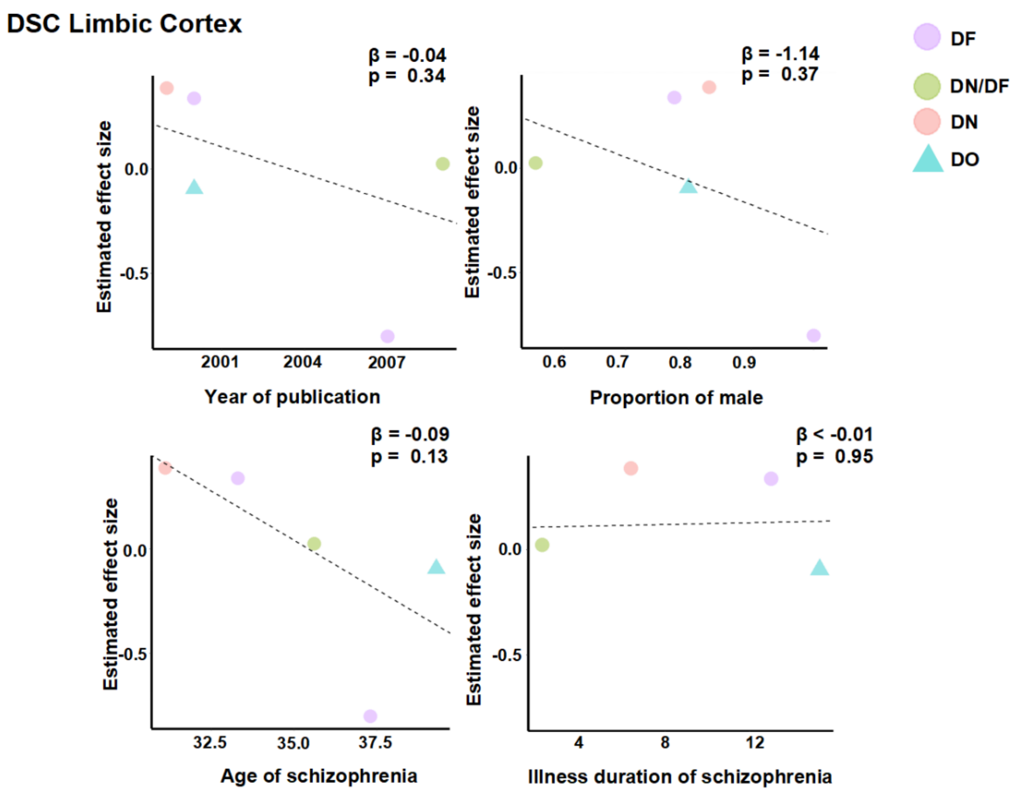

Supplementary Fig 6. Meta-regression with DSC. Publication year, proportion of male patients, patients' age, illness duration and severity of patient sympotoms in Limbic cortex DSC.

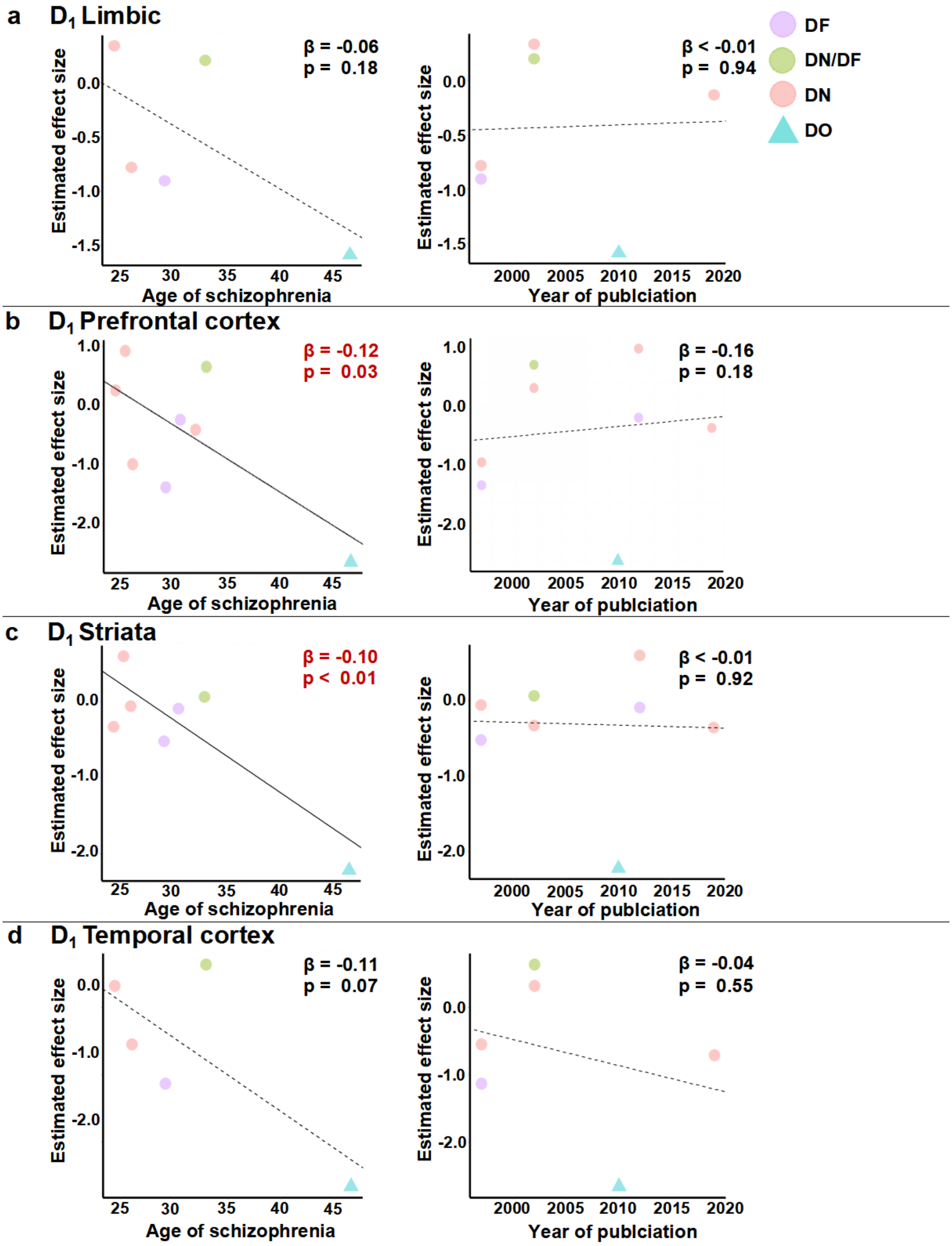

Supplementary Fig 7. Meta-regression with publication year and patients' age in D_1 receptor availability. Limbic cortex **(a)**, Prefrontal cortex **(b),** Striata **(b),**and Temporal cortex **(d)**,.

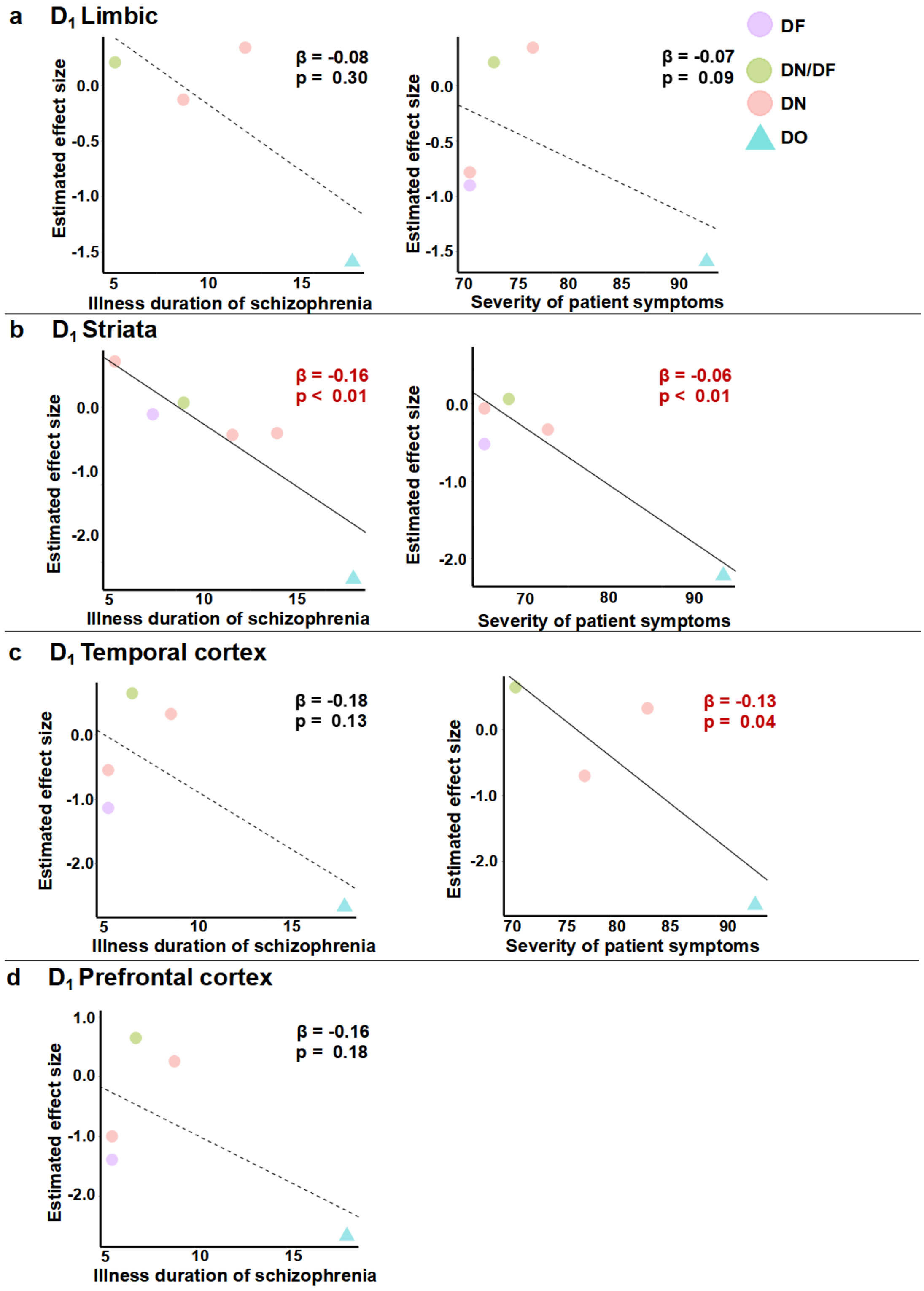

Supplementary Fig 8. Meta-regression with illness duration and severity of patient sympotoms in D_1 receptor availability. Limbic cortex **(a)**, Striata **(b),** Temporal cortex **(c),** and Prefrontal cortex **(d)**.

Uncategorized References

1 Wong, D. F. *et al.* Positron emission tomography reveals elevated D2 dopamine receptors in drug-naive schizophrenics. *Science* **234**, 1558-1563 (1986). https://doi.org:10.1126/science.2878495

2 Farde, L. *et al.* D2 dopamine receptors in neuroleptic-naive schizophrenic patients. A positron emission tomography study with [11C]raclopride. *Arch Gen Psychiatry* **47**, 213-219 (1990). https://doi.org:10.1001/archpsyc.1990.01810150013003

3 Tune, L. E. *et al.* Dopamine D2 receptor density estimates in schizophrenia: a positron emission tomography study with 11C-N-methylspiperone. *Psychiatry Res* **49**, 219-237 (1993). https://doi.org:10.1016/0165-1781(93)90063-m

4 Hietala, J. *et al.* Striatal D2 dopamine receptor characteristics in neuroleptic-naive schizophrenic patients studied with positron emission tomography. *Arch Gen Psychiatry* **51**, 116-123 (1994). https://doi.org:10.1001/archpsyc.1994.03950020040004

5 Nordström, A. L., Farde, L., Eriksson, L. & Halldin, C. No elevated D2 dopamine receptors in neuroleptic-naive schizophrenic patients revealed by positron emission tomography and [11C]N-methylspiperone. *Psychiatry Res* **61**, 67-83 (1995). https://doi.org:10.1016/0925-4927(95)02732-d

6 Okubo, Y. *et al.* Decreased prefrontal dopamine D1 receptors in schizophrenia revealed by PET. *Nature* **385**, 634-636 (1997). https://doi.org:10.1038/385634a0

7 Breier, A. *et al.* Schizophrenia is associated with elevated amphetamine-induced synaptic dopamine concentrations: evidence from a novel positron emission tomography method. *Proc Natl Acad Sci U S A* **94**, 2569-2574 (1997). https://doi.org:10.1073/pnas.94.6.2569

8 Suhara, T. *et al.* Decreased dopamine D2 receptor binding in the anterior cingulate cortex in schizophrenia. *Arch Gen Psychiatry* **59**, 25-30 (2002). https://doi.org:10.1001/archpsyc.59.1.25

9 Talvik, M., Nordström, A. L., Olsson, H., Halldin, C. & Farde, L. Decreased thalamic D2/D3 receptor binding in drug-naive patients with schizophrenia: a PET study with [11C]FLB 457. *Int J Neuropsychopharmacol* **6**, 361-370 (2003). https://doi.org:10.1017/s1461145703003699

10 Yasuno, F. *et al.* Low dopamine d(2) receptor binding in subregions of the thalamus in schizophrenia. *Am J Psychiatry* **161**, 1016-1022 (2004). https://doi.org:10.1176/appi.ajp.161.6.1016

11 Gründer, G. *et al.* The striatal and extrastriatal D2/D3 receptor-binding profile of clozapine in patients with schizophrenia. *Neuropsychopharmacology* **31**, 1027-1035 (2006). https://doi.org:10.1038/sj.npp.1300931

12 Buchsbaum, M. S. *et al.* D2/D3 dopamine receptor binding with [F-18]fallypride in thalamus and cortex of patients with schizophrenia. *Schizophr Res* **85**, 232-244 (2006). https://doi.org:10.1016/j.schres.2006.03.042

13 Talvik, M. *et al.* Dopamine D2 receptor binding in drug-naïve patients with schizophrenia examined with raclopride-C11 and positron emission tomography. *Psychiatry Res* **148**, 165-173 (2006). https://doi.org:10.1016/j.pscychresns.2006.05.009

14 Graff-Guerrero, A. *et al.* The effect of antipsychotics on the high-affinity state of D2 and D3 receptors: a positron emission tomography study With [11C]-(+)-PHNO. *Arch Gen Psychiatry* **66**, 606-615 (2009). https://doi.org:10.1001/archgenpsychiatry.2009.43

15 Graff-Guerrero, A. *et al.* The dopamine D2 receptors in high-affinity state and D3 receptors in schizophrenia: a clinical [11C]-(+)-PHNO PET study. *Neuropsychopharmacology* **34**, 1078-1086 (2009). https://doi.org:10.1038/npp.2008.199

16 Kessler, R. M. *et al.* Dopamine D2 receptor levels in striatum, thalamus, substantia nigra, limbic regions, and cortex in schizophrenic subjects. *Biol Psychiatry* **65**, 1024-1031 (2009). https://doi.org:10.1016/j.biopsych.2008.12.029

17 Kegeles, L. S. *et al.* Striatal and extrastriatal dopamine D2/D3 receptors in schizophrenia evaluated with [18F]fallypride positron emission tomography. *Biol Psychiatry* **68**, 634-641 (2010). https://doi.org:10.1016/j.biopsych.2010.05.027

18 Kegeles, L. S. *et al.* Increased synaptic dopamine function in associative regions of the striatum in schizophrenia. *Arch Gen Psychiatry* **67**, 231-239 (2010). https://doi.org:10.1001/archgenpsychiatry.2010.10

19 Slifstein, M. *et al.* Deficits in prefrontal cortical and extrastriatal dopamine release in schizophrenia: a positron emission tomographic functional magnetic resonance imaging study. *JAMA Psychiatry* **72**, 316-324 (2015). https://doi.org:10.1001/jamapsychiatry.2014.2414

20 Nakajima, S. *et al.* Dopamine $D_{2/3}$ receptor availability in the striatum of antipsychotic-free older patients with schizophrenia-A [^{11}C]-raclopride PET study. *Schizophr Res* **164**, 263-267 (2015). https://doi.org:10.1016/j.schres.2015.02.020

21 Joo, Y. H. *et al.* The relationship between excitement symptom severity and extrastriatal dopamine D(2/3) receptor availability in patients with schizophrenia: a high-resolution PET study with [(18)F]fallypride. *Eur Arch Psychiatry Clin Neurosci* **268**, 529-540 (2018). https://doi.org:10.1007/s00406-017-0821-y

22 Frankle, W. G. *et al.* Amphetamine-Induced Striatal Dopamine Release Measured With an Agonist Radiotracer in Schizophrenia. *Biol Psychiatry* **83**, 707-714 (2018). https://doi.org:10.1016/j.biopsych.2017.11.032

23 Veselinović, T. *et al.* The role of striatal dopamine D(2/3) receptors in cognitive performance in drug-free patients with schizophrenia. *Psychopharmacology (Berl)* **235**, 2221-2232 (2018). https://doi.org:10.1007/s00213-018-4916-6

24 Schifani, C. *et al.* Cortical stress regulation is disrupted in schizophrenia but not in clinical high risk for psychosis. *Brain* **141**, 2213-2224 (2018). https://doi.org:10.1093/brain/awy133

25 Plavén-Sigray, P. *et al.* Thalamic dopamine D2-receptor availability in schizophrenia: a study on antipsychotic-naive patients with first-episode psychosis and a meta-analysis. *Mol Psychiatry* **27**, 1233-1240 (2022). https://doi.org:10.1038/s41380-021-01349-x
26 Hietala, J. *et al.* Presynaptic dopamine function in striatum of neuroleptic-naive schizophrenic patients. *Lancet* **346**, 1130-1131 (1995). https://doi.org:10.1016/s0140-6736(95)91801-9
27 Dao-Castellana, M. H. *et al.* Presynaptic dopaminergic function in the striatum of schizophrenic patients. *Schizophr Res* **23**, 167-174 (1997). https://doi.org:10.1016/s0920-9964(96)00102-8
28 Lindström, L. H. *et al.* Increased dopamine synthesis rate in medial prefrontal cortex and striatum in schizophrenia indicated by L-(beta-11C) DOPA and PET. *Biol Psychiatry* **46**, 681-688 (1999). https://doi.org:10.1016/s0006-3223(99)00109-2
29 Hietala, J. *et al.* Depressive symptoms and presynaptic dopamine function in neuroleptic-naive schizophrenia. *Schizophr Res* **35**, 41-50 (1999). https://doi.org:10.1016/s0920-9964(98)00113-3
30 Elkashef, A. M. *et al.* 6-(18)F-DOPA PET study in patients with schizophrenia. Positron emission tomography. *Psychiatry Res* **100**, 1-11 (2000). https://doi.org:10.1016/s0925-4927(00)00064-0
31 McGowan, S., Lawrence, A. D., Sales, T., Quested, D. & Grasby, P. Presynaptic dopaminergic dysfunction in schizophrenia: a positron emission tomographic [18F]fluorodopa study. *Arch Gen Psychiatry* **61**, 134-142 (2004). https://doi.org:10.1001/archpsyc.61.2.134
32 Kumakura, Y. *et al.* Elevated [18F]fluorodopamine turnover in brain of patients with schizophrenia: an [18F]fluorodopa/positron emission tomography study. *J Neurosci* **27**, 8080-8087 (2007). https://doi.org:10.1523/jneurosci.0805-07.2007
33 Nozaki, S. *et al.* Regional dopamine synthesis in patients with schizophrenia using L-[beta-11C]DOPA PET. *Schizophr Res* **108**, 78-84 (2009). https://doi.org:10.1016/j.schres.2008.11.006
34 Howes, O. D. *et al.* Elevated striatal dopamine function linked to prodromal signs of schizophrenia. *Arch Gen Psychiatry* **66**, 13-20 (2009). https://doi.org:10.1001/archgenpsychiatry.2008.514
35 Demjaha, A., Murray, R. M., McGuire, P. K., Kapur, S. & Howes, O. D. Dopamine synthesis capacity in patients with treatment-resistant schizophrenia. *Am J Psychiatry* **169**, 1203-1210 (2012). https://doi.org:10.1176/appi.ajp.2012.12010144
36 Jauhar, S. *et al.* A Test of the Transdiagnostic Dopamine Hypothesis of Psychosis Using Positron Emission Tomographic Imaging in Bipolar Affective Disorder and Schizophrenia. *JAMA Psychiatry* **74**, 1206-1213 (2017). https://doi.org:10.1001/jamapsychiatry.2017.2943
37 Kim, E. *et al.* Presynaptic Dopamine Capacity in Patients with Treatment-Resistant Schizophrenia Taking Clozapine: An [(18)F]DOPA PET Study. *Neuropsychopharmacology* **42**, 941-950 (2017). https://doi.org:10.1038/npp.2016.258
38 Jauhar, S. *et al.* Determinants of treatment response in first-episode psychosis: an (18)F-DOPA PET study. *Mol Psychiatry* **24**, 1502-1512 (2019). https://doi.org:10.1038/s41380-018-0042-4

39 Avram, M. *et al.* Reduced striatal dopamine synthesis capacity in patients with schizophrenia during remission of positive symptoms. *Brain* **142**, 1813-1826 (2019). https://doi.org:10.1093/brain/awz093

40 Abi-Dargham, A. *et al.* Prefrontal dopamine D1 receptors and working memory in schizophrenia. *J Neurosci* **22**, 3708-3719 (2002). https://doi.org:10.1523/jneurosci.22-09-03708.2002

41 Karlsson, P., Farde, L., Halldin, C. & Sedvall, G. PET study of D(1) dopamine receptor binding in neuroleptic-naive patients with schizophrenia. *Am J Psychiatry* **159**, 761-767 (2002). https://doi.org:10.1176/appi.ajp.159.5.761

42 Kosaka, J. *et al.* Decreased binding of [11C]NNC112 and [11C]SCH23390 in patients with chronic schizophrenia. *Life Sci* **86**, 814-818 (2010). https://doi.org:10.1016/j.lfs.2010.03.018

43 Abi-Dargham, A. *et al.* Increased prefrontal cortical D_1 receptors in drug naive patients with schizophrenia: a PET study with [^{11}C]NNC112. *J Psychopharmacol* **26**, 794-805 (2012). https://doi.org:10.1177/0269881111409265

44 Stenkrona, P., Matheson, G. J., Halldin, C., Cervenka, S. & Farde, L. D1-Dopamine Receptor Availability in First-Episode Neuroleptic Naive Psychosis Patients. *Int J Neuropsychopharmacol* **22**, 415-425 (2019). https://doi.org:10.1093/ijnp/pyz017

45 Laakso, A. *et al.* Striatal dopamine transporter binding in neuroleptic-naive patients with schizophrenia studied with positron emission tomography. *Am J Psychiatry* **157**, 269-271 (2000). https://doi.org:10.1176/appi.ajp.157.2.269

46 Laakso, A. *et al.* Decreased striatal dopamine transporter binding in vivo in chronic schizophrenia. *Schizophr Res* **52**, 115-120 (2001). https://doi.org:10.1016/s0920-9964(00)00095-5

47 Arakawa, R. *et al.* Increase in thalamic binding of [(11)C]PE2I in patients with schizophrenia: a positron emission tomography study of dopamine transporter. *J Psychiatr Res* **43**, 1219-1223 (2009). https://doi.org:10.1016/j.jpsychires.2009.04.009

48 Artiges, E. *et al.* Striatal and Extrastriatal Dopamine Transporter Availability in Schizophrenia and Its Clinical Correlates: A Voxel-Based and High-Resolution PET Study. *Schizophr Bull* **43**, 1134-1142 (2017). https://doi.org:10.1093/schbul/sbw192
